# Fertile interspecific diploid hybrids between the Asian and African rice species facilitated by tetraploidization and its reduction

**DOI:** 10.1007/s00122-025-04901-3

**Published:** 2025-06-27

**Authors:** Daichi Kuniyoshi, Yuji Kishima

**Affiliations:** 1 Tropical Agriculture Research Front, Japan International Research Center for Agricultural Sciences, Ishigaki, 305-8686 Japan; 2 Laboratory of Plant Breeding, Research Faculty of Agriculture, Hokkaido University, Sapporo, 060-0808 Japan

## Abstract

**Key message:**

We developed fertile diploid interspecific hybrids that were genetically balanced between the Asian rice, *Oryza sativa,* and the African rice, *Oryza glaberrima*, through tetraploidization and subsequent diploid induction.

**Abstract:**

Interspecific hybrids between the Asian rice, *Oryza sativa,* and the African rice, *O. glaberrima,* show severe pollen grain sterility owing to the sterility effect of multiple hybrid sterility (HS) genes/loci. These HS loci only cause pollen grain sterility in heterozygotic situations; therefore, interspecific hybrids can be made fertile by fixing all HS loci as homozygous if the hybrids inherit the genomes from both species equally. Such genetically balanced hybrids can combine the superior traits of both species. However, a method for developing balanced hybrids with fixed HS loci is lacking. Previously, a diploid interspecific hybrid population was obtained through anther culture of tetraploid interspecific hybrids, and in this study, 22 double haploid (DH) plants were developed through anther culture of the diploid interspecific hybrids. The DH plants were genetically fixed, including the HS loci, and confirmed to be genetically balanced between the two species. Nine of the DH plants showed a pollen fertility of more than 60%, and the progeny DH lines developed through self-pollination of the DH plants varied phenotypically for each line. These results demonstrate that genetically balanced hybrids between *O. sativa* and *O. glaberrima* with fixed HS loci can be developed through successive anther cultures of tetraploid interspecific hybrids. The balanced hybrids maintained the genome of *O. glaberrima* at higher ratios than traditional backcrossing varieties. Therefore, this breeding strategy using tetraploid hybrids as intermediators for the development of balanced diploid hybrids will provide new interspecific varieties that combine the superior traits of both species.

**Supplementary Information:**

The online version contains supplementary material available at 10.1007/s00122-025-04901-3.

## Introduction

*Oryza sativa* (the Asian rice) and *O. glaberrima* (the African rice) can easily be hybridized by crossing; however, as the interspecific F_1_ hybrids of the two species show hybrid sterility, especially in pollen production, progeny generation cannot be obtained through self-pollination. The main cause of hybrid sterility is the hybrid sterility genes/loci (HS loci), which induce sterility in gametes. Multiple HS loci with more than 15 loci were identified between *O. sativa* and *O. glaberrima* as follows: *S1*, *S2*, *S3*, *S18*, *S19*, *S20*, *S21*, *S29(t)*, *S33(t)*, *S34(t)*, *S36(t)*, *S37(t)*, *S38(t)*, *S39(t)*, and *S58* (Sano et al. 1979; Sano 1983; Doi et al. 1998, 1999; Ren et al. 2005; Zhang et al. 2005, 2011; Hu et al. 2006; Li et al. 2011; Xu et al. 2014; Wang et al. 2020; Feng et al. 2023; Myint et al. 2024). All of these HS loci cause pollen grain sterility, but only a few cause sterility in female gametes; thus, interspecific F_1_ hybrids show complete sterility of pollen grains and partial fertility of the ovules.

Furthermore, these HS loci cause sterility only when they are maintained in a heterozygous state for two different alleles, one from *O. sativa* and the other from *O. glaberrima,* in hybrid plants (Xie et al. 2019a). In addition, except for the *S18* locus, these HS loci affect only the gametes with a recessive type of allele to become sterile. For example, in plants carrying heterozygous alleles such as *S1-s* from *O. sativa* and *S1-g* from *O. glaberrima*, gametes carrying the *S1-s* allele will be sterile, whereas only gametes carrying *S1-g* are fertile (Sano et al. 1979; Koide et al. 2008). Thus, 50% of the total gametes would be sterile owing to the effect of a single HS locus, and interspecific F_1_ hybrids would be fully pollen-sterile owing to the accumulation of multiple HS loci.

The most commonly observed system for HS loci across *Oryza* species is the killer–protector (KP) mechanism (Yang et al. 2012; Yu et al. 2018; Xie et al. 2019b; Wang et al. 2023; You et al. 2023). The *S1* and possibly *S21* loci between *O. sativa* and *O. glaberrima* operate under this mechanism (Yu et al. 2018; Xie et al. 2019b; Li et al. 2020). In this model, alleles that preferentially transmit encode both the killer and protector factors, which are tightly linked to each other (referred to as the KP allele). The killer induces sterility of gametes sporophytically, whereas the protector eliminates the killer effect gametophytically (Fig. S1). The recessive alleles, which do not encode both factors, are referred to as the killed allele (Fig. S1). Therefore, only gametes carrying the killed allele become sterile in plants with a heterozygous HS locus via the KP mechanism (Fig. S1).

The *Hybrid Weakness and Sterility* (*HWS*) genes have recently been identified as the cause of sterility in interspecific hybrids of *O. sativa* and *O. glaberrima*, apart from the HS loci (Liao et al. 2024). Two orthologous *HWS* genes exist: *HWS1* on chromosome 1 and *HWS2* on chromosome 12. *HWS1* from *O. glaberrima* and *HWS2* from *O. sativa* retained their functions, and at least one of the *HWS* genes is necessary for anther and pollen development (Liao et al. 2024). Interspecific F_1_ hybrids will not be affected by this *HWS*-based sterility because F_1_ hybrids have the *HWS* genes, but the progeny generation of the two species will be sterile if they lose both functional *HWS* genes (Liao et al. 2024). Therefore, the HS loci are the main causal factor for hybrid sterility in the interspecific F_1_ hybrids.

Severe sterility of pollen grains hinders the self-pollination of interspecific F_1_ hybrids; thus, the use of *O. glaberrima* germplasms is performed through backcrossing using *O. sativa* as the recurrent parent, with the development of several series of chromosome segment substitution lines (CSSL) or backcross-inbred lines (Ali et al. 2010; Garavito et al. 2010; Bimpong et al. 2011; Yamagata et al. 2019). These CSSLs have chromosomal segments from *O. glaberrima* with the genetic background of *O. sativa*, which allows the combination of the high-yielding ability and good eating quality of *O. sativa* varieties and useful traits in *O. glaberrima* that are controlled by a single or a few genes, such as resistance for pathogens or tolerance for abiotic stresses (Majerus et al. 2007; Dong et al. 2020; Pidon et al. 2020). The upland NERICA series is a notable CSSL variety between *O. sativa* and *O. glaberrima*, which was bred by WARDA in the 1990s and cultivated on approximately 200,000 ha in multiple sub-Saharan African countries (Somado et al. 2008).

The use of *O. glaberrima* germplasms through successive backcrossing is a reliable and successful method to improve the qualitative traits of *O. sativa* varieties. However, the CSSL varieties do not exhibit several useful traits that were expected to be inherited from *O. glaberrima*, such as weed competitiveness and adaptability to low soil fertility (Saito et al. 2012). These traits might be quantitative traits controlled by multiple genetic factors and not by a single or few genes; thus, more genetic segments would be required to incorporate those traits into the varieties. The development of recombinant inbred lines (RIL) or doubled haploid (DH) lines, which have genetically balanced genomes from both species, is a more effective method to use these traits. However, the production of genetically balanced hybrids, such as RILs, is impossible because interspecific F_1_ hybrids are pollen-sterile.

In rice, DH plants are usually induced from haploid gametes via microspores or anther cultures (Tripathy et al. 2019). Because DH plants consist of homozygous alleles, they are unaffected by HS loci, which cause conflicts between heterozygous alleles. Thus, the DH lines of interspecific hybrids are theoretically fertile even when the two species have antagonistic genomic compositions. Based on this assumption, an attempt was previously made to produce diploid DH plants through anther culture of the interspecific F_1_ hybrids between the two species; however, this attempt was unsuccessful because of pollen grain sterility in the F_1_ hybrid (Kanaoka et al. 2018; Kuniyoshi et al. 2020).

**Fig. 1:**
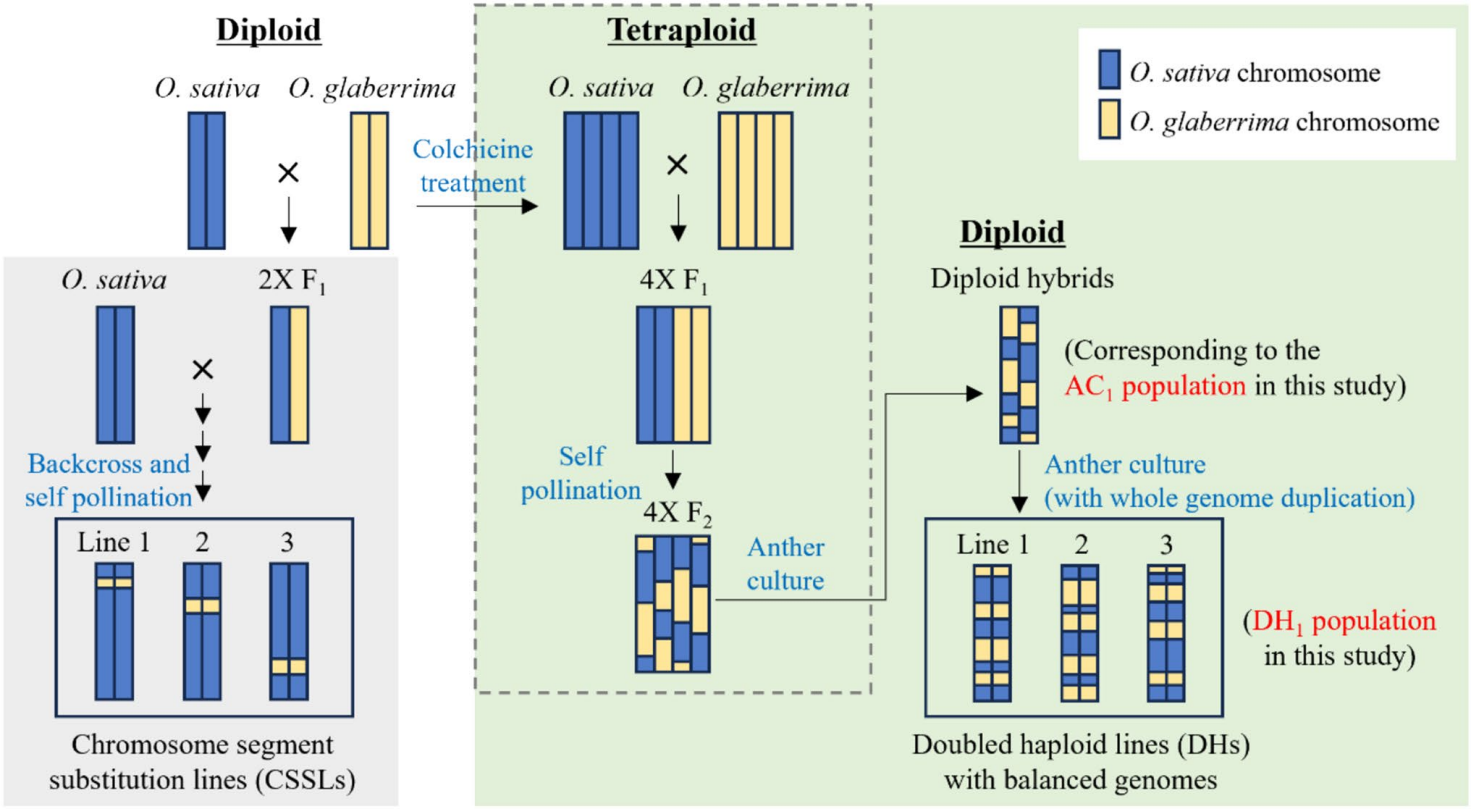
Genomic differences between the chromosome segment substitution lines and doubled haploid lines genetically balanced between the two species. The diploid hybrids and double haploid lines corresponded to the AC_1_ and DH_1_ populations in the present study

Although fertile diploid DH plants were not obtained, tetraploid hybrids carrying multiple HS loci in a heterozygous state were obtained, and these plants exhibited partial pollen fertility (Kuniyoshi et al. 2020). Based on these results, the tetraploid interspecific F_1_ hybrids between *O. sativa* and *O. glaberrima* were further studied, and it was demonstrated that the F_1_ hybrids could restore pollen fertility in tetraploidy by reducing the sterility effect of the HS locus with the KP mechanism (Fig. S1) (Kuniyoshi et al. 2024). Additionally, interspecific diploid hybrids were obtained through anther culture of a tetraploid hybrid (WK21-4X/PL9-4X F_2_) population (Kuniyoshi et al. 2024). These diploid interspecific hybrids (hereafter referred to as the AC_1_ population) were partially genetically fixed, especially in the genetic regions where the HS loci were located (Fig. 1) (Kuniyoshi et al. 2024). Therefore, these diploid hybrids are expected to partially restore pollen fertility and can be used for anther culture to successfully produce interspecific DH plants.

In the present study, it is demonstrated that tetraploid interspecific hybrids mediate genetic exchange between the two species by decreasing the sterility effect of the HS loci, and fertile diploid hybrids with genetically balanced genomes between the two species were obtained by anther culturing of the tetraploid hybrids. The genomic composition was also determined and the phenotypic diversity of the DH plants as balanced hybrids was evaluated.

## Materials and methods

### Plant materials

PL9-2X of *O. sativa* ssp. *japonica* and WK21-2X of *O. glaberrima* were used as parental lines. In our previous study (Kuniyoshi et al. 2024), diploid interspecific hybrids (the AC_1_ population) between two parents were produced through anther culture of tetraploid WK21-4X/PL9-4X F_2_ hybrids (Fig. 1). The AC_1_ population consisted of 134 diploid hybrids with partially fixed genomes. The AC_1_ plants were used to produce the DH plants (referred to as the DH_1_ population). WK21-2X/PL9-2X F_1_ hybrids were generated by crossing two parents and were used for pollen observation.

### Pollen observation

Pollen was collected during the flowering stage. Spikelets containing mature pollen were removed from panicles and collected in 0.5 mL tubes containing FAA fixative (50% ethanol, 5% acetic acid, and 5% formaldehyde). Anthers were excised from the spikelets, placed in a new 0.5 mL tube with 20 µL of I_2_-KI solution, chopped using tweezers, and observed using an optical microscope, BX43 (EVIDENT). All plant materials were grown in a paddy field at the Tropical Agriculture Research Front of the Japan International Research Center for Agricultural Sciences, Ishigaki, Okinawa, Japan.

### Anther culture

Diploid interspecific hybrids in the AC_1_ population were used for anther cultures to produce DH plants with fully fixed genomes (Fig. 1). Among the 134 individuals in the AC_1_ population, 90 were used for anther culture. The other culture method was similar to that previously described (Kuniyoshi et al. 2024). Panicles were removed from the plants at the booting stage, when the distance between the auricles of the flag and penultimate leaves was 0–10 cm. They were then surface-sterilized with 70% ethanol and stored at 10 °C in dark conditions for 8–12 days. Anthers were excised from the spikelets and placed on an N6 callus induction medium in plastic dishes (GD90-15, AS ONE). The medium containing the anthers was kept at 25 °C under dark conditions and cultured for 2–3 months. Calli that emerged from the anthers were transferred to N6 regeneration media to induce regeneration and further cultured under the light at 25 °C. The regenerated plants were grown on regeneration media until they reached a height of approximately 10 cm and then were transplanted into soil.

### Ploidy analysis and genotyping of anther culture-derived plants

Anther cultures of diploid plants may produce regenerated plants derived from somatic cells (anther wall cells) or unreduced microspores with diploid genomes, in addition to the desired DH plants from haploid microspores. Furthermore, tetraploid DH plants could be regenerated from haploid microspores by duplicating the genome twice during culture. Therefore, ploidy analysis and genotyping of anther culture-derived plants were performed to select diploid DH plants with a fully fixed genome.

Ploidy analysis was conducted using a flow cytometer (Partec PA; Partec GmbH). Small leaf pieces from the plants were finely sliced in a nuclear extraction buffer (component A, Quantum Stain NA 2A, CytoTechs) using a razor blade. The mixture of sliced leaves and released nuclei was filtered using a 30 µm mesh (Partec CellTrics). Subsequently, a DAPI solution (Component B, Quantum Stain NA 2A, CytoTechs) was added to the filtrate containing nuclei. Nuclear suspensions were analyzed with a flow cytometer (Partec PA) to measure the fluorescence intensity of nuclei stained with DAPI. The ploidy level was determined by comparing the fluorescence intensity of the AC-derived plants with that of the diploid and tetraploid parental lines.

Among the 102 plants produced through anther culture of the AC_1_ population, 65 individuals were confirmed to be diploid, and 41 successfully grew into mature plants. To select DH plants, PCR-based genotyping of these plants was performed to confirm whether their genome was entirely fixed using randomly selected 24 DNA markers (Kuniyoshi et al. 2020, 2024). The PCR mixture comprised 5 µL of Taq master mix (Quick Taq HS Dye MIX, TOYOBO), 2 µL each of forward and reverse primers, and 1 µL of DNA templates. PCR amplification was performed using the following program settings: 95 °C for 3 min; 35 cycles of 95 °C for 30 s; 55 °C for 30 s; 72 °C for 30 s; 72 °C for 5 min; and 10 °C for storage. Electrophoresis was performed using gels containing 3% agarose (Agarose X, NIPPON GENE) and DNA staining (Midori Green Advance, Nippon Genetics) for 30–45 min. Based on genotyping, 34 of the 41 plants were confirmed as diploid DH plants.

### Genetic characterization by GRAS-Di analysis

Among the 34 diploid DH plants produced through anther culture of the AC_1_ population, several regenerated from the same callus. These individuals shared the same genome; therefore, only one DH plant was selected from each callus, and 22 diploid DH plants were selected, resulting in a DH population (hereafter DH_1_ population) (Fig. 1).

The genomic composition of DH plants in the DH_1_ population was analyzed using GRAS-Di analysis (Seibutsugiken Co., Ltd.), a whole-genome genotyping service. The genomic DNA of DH plants was extracted from leaf pieces using a DNeasy Plant Mini Kit (QIAGEN), and the DNA samples were subjected to GRAS-Di analysis. Raw read files (fastq format; raw files are deposited in the DDBJ BioProject database with BioProject accession number PRJDB19059), including the sequence data of the DH materials, were processed according to the method described by Dinh et al. (2023). The quality of the raw reads in the file was checked using FastQC software (ver. 0.12.1, https://www.bioinformatics.babraham.ac.uk/projects/fastqc/). The raw reads were trimmed using Trimmomatic software (ver. 0.36) with the settings LEADING:3, TRAILING:3, SLIDINGWINDOW:4:15, and MINLEN:100 (Bolger et al. 2014). The reads were then aligned to the Nipponbare genome sequence (IRGSP 1.0, https://rapdb.dna.affrc.go.jp/download/irgsp1.html) using BWA software (ver. 0.7.17) (Li and Durbin 2009). The reads were sorted and converted to the BAM format using SAMtools software (ver. 1.9) (Li et al. 2009). The reads in the BAM files were screened based on their mapping score with a threshold of 60 and then indexed using SAMtools software. Variant calling for mapped reads was performed using the bcftools software (ver. 1.8) (Li et al. 2009). To prevent incorrectly defining genotypes as heterozygous, genotypes in polymorphic regions were defined as heterozygous only if the number of reads from each parent was greater than five. The vcf files were further filtered using the vcftools software (ver. 0.2.26) with settings of –max-missing 0.95, –minQ 20, –min-alleles 2, and –max-alleles 2 (Danecek et al. 2011).

To construct graphical genotype images of the DH plants, the genotype information in the vcf files was filtered using TASSEL software (ver. 5.2.93) with the settings of Site Min Allele Fre 0.01 and Max Heterozygous Proportion 0.99 (Bradbury et al. 2007). The genotype information was then screened based on its physical position using the settings of Thin Sites by Positions 100,000 bp to maintain equal distances between genotyped sites. The screened genotype information of the DH plants was then converted to the ABH genotype, where genotypes A and B represented the homozygous genotypes of PL9-2X and WK21-2X, respectively, and H was the heterozygous genotype of the two parents, using the ABH genotype option in TASSEL. Finally, graphical genotype images were constructed using Flapjack software (ver. 1.22.04.21), and an image of the frequency of the *sativa* allele across all chromosomes was constructed by R software (ver. 4.3.1), using the circlize package (ver. 0.4.16) (Milne et al. 2010).

### Phenotypic evaluation

Of the 22 individuals in the DH_1_ population, 10 successfully developed a progeny population. The agronomic traits of the 10 DH lines as well as PL9-2X and WK21-2X were evaluated. Seeds of the plant materials were sown on sterilized soil and grown for 1 month in a greenhouse; then, the seedlings were transplanted in a paddy field at the Tropical Agriculture Research Front of the Japan International Research Center for Agricultural Sciences, Ishigaki, Okinawa, Japan (24.20°N, 124.09°E). Ten plants per material were planted in a single row, with one plant per hill, spaced 18 cm apart, and each row was spaced 30 cm apart. Among the 10 plants, excluding the individuals located at the edge of the row, three plants with average growth were selected for phenotypic evaluation. The following nine traits were evaluated: culm length (CL), panicle length (PL), number of panicles per plant (PN), dried culm weight (CW), dried panicle weight (PW), harvest index (HI), number of spikelets per single panicle (TS), weight of one thousand grains (TGW), and number of days from sowing to heading (DTH). HI and TGW were calculated as follows: $$\text{HI}\hspace{0.17em}=\hspace{0.17em}\frac{PW}{CW+PW}$$, and $$\text{TGW}\hspace{0.17em}=\hspace{0.17em}\frac{Weight of fertile seeds in a panicle}{No. of the fertile seeds}\times 1000$$. Except for DTH, the evaluation was performed after harvesting, and the materials were thoroughly dried. DTH was defined as the average of the days until the first individual headed, until half of the individuals headed, and until the last individual headed.

Statistical analysis was performed using the nonparametric Kruskal–Wallis test. The significance threshold was set at *p* < 0.05. Multiple pairwise comparisons were performed using Dunn’s test, and the p-values were then adjusted to q-values using the Benjamini–Hochberg (BH) adjustment. The significance threshold was set at q < 0.05.

In addition to evaluating these agronomic traits, pollen fertility (PF) and seed set rate (SR) were observed in the progeny generation to confirm that fertility is stably inherited. SR was calculated as follows: $\frac{No.offertilespikelets}{No.oftotal\left(fertile+sterile\right)spikelets}\times 100 \left(\%\right)$.

## Results

### Pollen fertility of parental lines and diploid hybrids

**Fig. 2:**
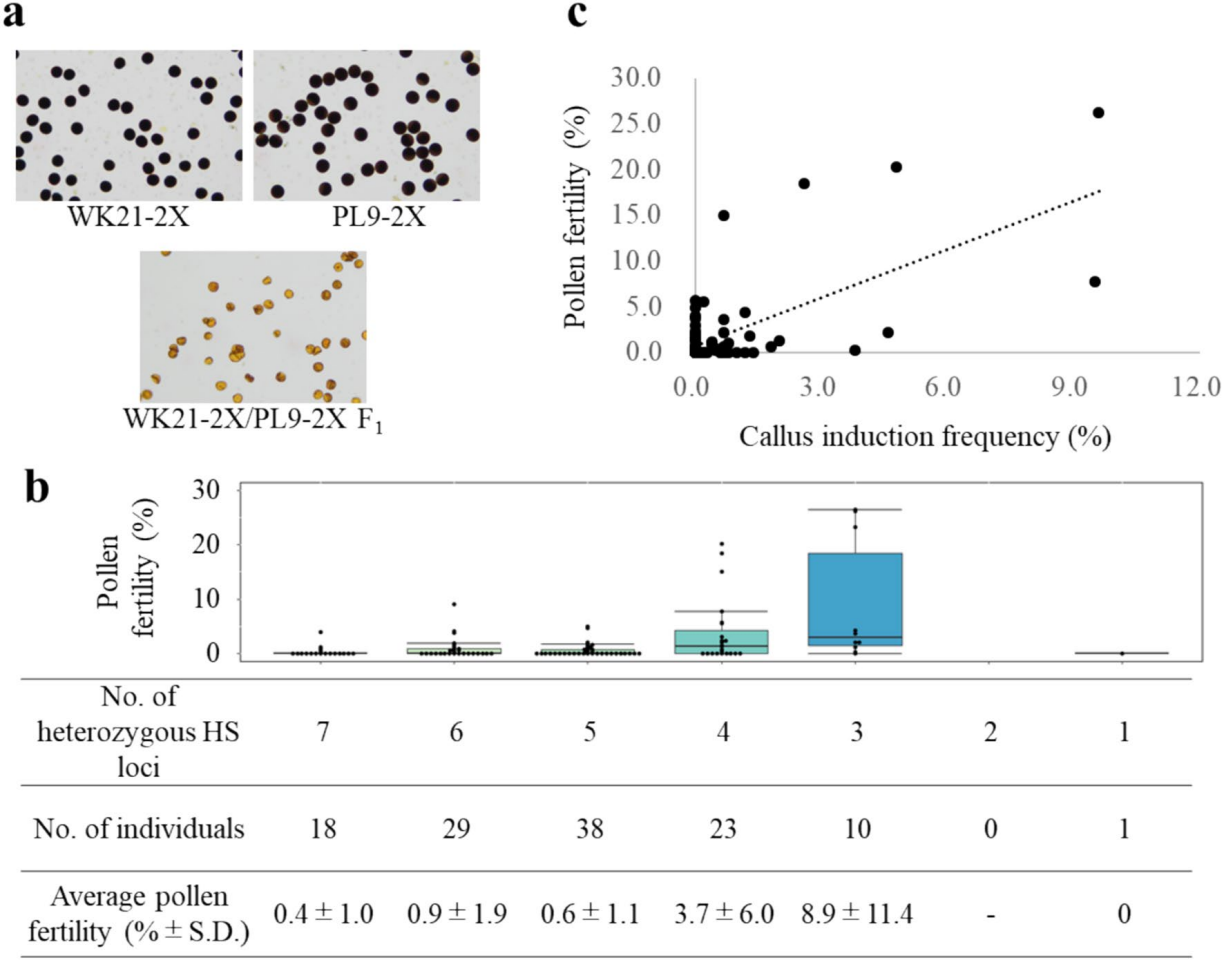
Pollen fertility of the parental lines, F_1_ hybrids, and the AC_1_ plants. **a** Pollen observation of the parental lines PL9-2X and WK21-2X and WK21-2X/PL9-2X F_1_ hybrids, stained with I_2_-KI staining. **b** Distribution of the pollen fertility of the diploid hybrid plants in the AC_1_ population. The plants were divided into six classes based on the number of heterozygous HS loci (1, 3–7) in each individual. The data on the number of heterozygous HS loci in the AC_1_ plants were sourced from Kuniyoshi et al. 2024. **c** Relationship between pollen fertility and callus induction frequency (CIF) in the AC_1_ population. CIF in the AC_1_ population tended to increase as the pollen fertility is increased (*R* = 0.66)

The parental lines PL9-2X and WK21-2X were confirmed to be pollen-fertile, whereas the interspecific WK21-2X/PL9-2X F_1_ hybrids showed severe sterility of pollen grains, as previously confirmed (Fig. 2a, Table 1) (Kuniyoshi et al. 2024).

**Table 1:** Pollen fertility of PL9-2X, WK21-2X and WK21/PL9-2X F_1_ hybrid, and diploid hybrids in AC_1_ population

Plant materials	Generation	No. of individuals	Pollen fertility (%)
PL9-2X	(Parent)	3	96.9 ± 1.0
WK21-2X		3	91.4 ± 1.7
WK21-2X/PL9-2X F_1_	F_1_	3	0.0 ± 0.0
AC_1_ population	AC_1_	119*	1.9 ± 4.9

*Pollen of 119 individuals out of total 134 individuals in the AC_1_ population was observed

In a previous study, tetraploid interspecific F_1_ hybrids between WK21-4X and PL9-4X were developed by crossing, and it was confirmed that the WK21-4X/PL9-4X F_1_ hybrid plants were pollen-fertile; thus, F_2_ seeds were successfully obtained (Kuniyoshi et al. 2024). A total of 134 diploid interspecific hybrids in the AC_1_ population were produced by anther culture of the WK21-4X/PL9-4X F_2_ hybrids, and pollen grain fertility of 119 individuals was successfully observed (Fig. 1). In the AC_1_ population, 55 individuals showed pollen fertility ranging from 0.2 to 26.4%, whereas 64 individuals were completely pollen-sterile (0.0%), similar to the WK21/PL9-2X F_1_ hybrids (Tables 1 and Table S1). All diploid hybrids in the AC_1_ population were segregated into three genotypes (*sativa* (*S*), *glaberrima* (*G*), and hetero (*H*)) when the 21 polymorphic sites were genotyped, whereas these loci were all heterozygous in the WK21-2X/PL9-2X F_1_ hybrid (Table S2).

The diploid hybrids in the AC_1_ population tended to restore their pollen fertility as the HS loci became fixed. The average pollen fertility of the 10 individuals carrying 3 of the 7 HS loci in a heterozygous state reached 8.9% (Fig. 2b; data on the number of heterozygous HS loci in the AC_1_ plants were sourced from Kuniyoshi et al. 2024). Pollen fertility and the number of heterozygous HS loci in the AC_1_ population were weakly correlated (*R* = −0.37) (Fig. S2, Table S1). These results suggest that diploid interspecific hybrids between the two species could be made pollen-fertile by fixing the HS loci.

### Anther culture of the AC_1_ population

The genomes of AC_1_ plants were partially fixed (Table S2), and pollen fertility was restored when the HS loci were fixed (Figs. 2b and S2). Anther culture was performed on 90 individuals of the AC_1_ population to produce DH plants. The callus induction frequencies of PL9-2X and WK21-2X were 6.2% and 3.8%, respectively, and 0.3% in the WK21-2X/PL9-2X F_1_ hybrids (Table 2). The average callus induction frequency in the AC_1_ population was 1.1%, with the highest frequency being 9.6% in plant AC#7, which was higher than those in both PL9-2X and WK21-2X (Tables 2 and Table S1). A strong positive correlation (*R* = 0.66) was observed between callus induction frequency and pollen fertility in the AC_1_ population (Fig. 2c, Table S1).

**Table 2:** Result of anther culture of parentala lines, WK21-2X/PL9-2X F_1_ plants and the AC_1_ plants

Plant materials	No. ofcultured anther (A)	No. of anthers producing calli (B)	Callus induction frequency (B/A %)	No. of replanted calli (C)	No. of regenerated calli (D)	No. of regenerated plants	Regeneration frequency ^*1^ (D/C %)
PL9-2X	226	14	6.2	14	6	6	42.9
WK21-2X	1298	49	3.8	30	3	3	10.0
WK21-2X/PL9-2X F_1_	588	2	0.3	2	0	0	0.0
AC_1_ population	34,141	389	1.1	351	75	102^*2^	21.4

^*1^The calli regenerated to green and sometimes albino plantlets also. Only the green plantlets were considered in this experiment, but the albino plantlets were ignored

^*2^Several calli formed two or more plantlets thus the number of the regenerated plants (102) were more than the number of the regenerated calli (75)

The regeneration frequencies from callus to green plantlets in PL9-2X and WK21-2X were 42.9% and 10.0%, respectively, whereas no regenerated plants were obtained from the WK21-2X/PL9-2X F_1_ hybrids (Table 2). In the AC_1_ population, 75 of 351 calli formed green plantlets, which were candidates for DH plants, with an average regeneration frequency of 21.4% (Table 2). Plant AC#60 in the AC_1_ population produced 21 regenerated plants, the highest number of regenerated plants in the AC_1_ population (Table S1). Although the total number of AC_1_-derived calli that produced regenerated plants was 75, multiple regenerated plants were produced from a single callus; therefore, finally, 102 regenerated plants were obtained from the 75 calli as candidates for diploid DH plants (Table 2).

Among the 102 regenerated plants of the diploid DH candidates, 65 individuals were confirmed to be diploid, and 41 successfully grew into mature plants (Tables 3 and S3). The genomes of these 41 diploid plants were analyzed for doubled haploid status by PCR-based genotyping using 24 polymorphic markers across the genome (Table S4). Of the 41 individuals, 34 were homozygous in all genotyped regions, indicating that these individuals were diploid DH plants (Table S5). Because several individuals among the 34 DH plants appeared from the same callus, these plants could share the same genome structure. Therefore, DH plants with unique genomes derived from different calli were chosen; 22 individuals were selected as diploid DH plants. These 22 DH plants were named the DH_1_ population, and their pollen fertility and genomic composition were analyzed.

**Table 3:** Ploidy of the anther culture plants derived from AC_1_ plants

Ploidy	No. of plants
Haploid	14
Diploid	65
Triploid	3
Tetraploid	17
Hexaploid	2
Octoploid	1
Total	102

### Pollen and seed fertility in the DH_1_ population

**Fig. 3:**
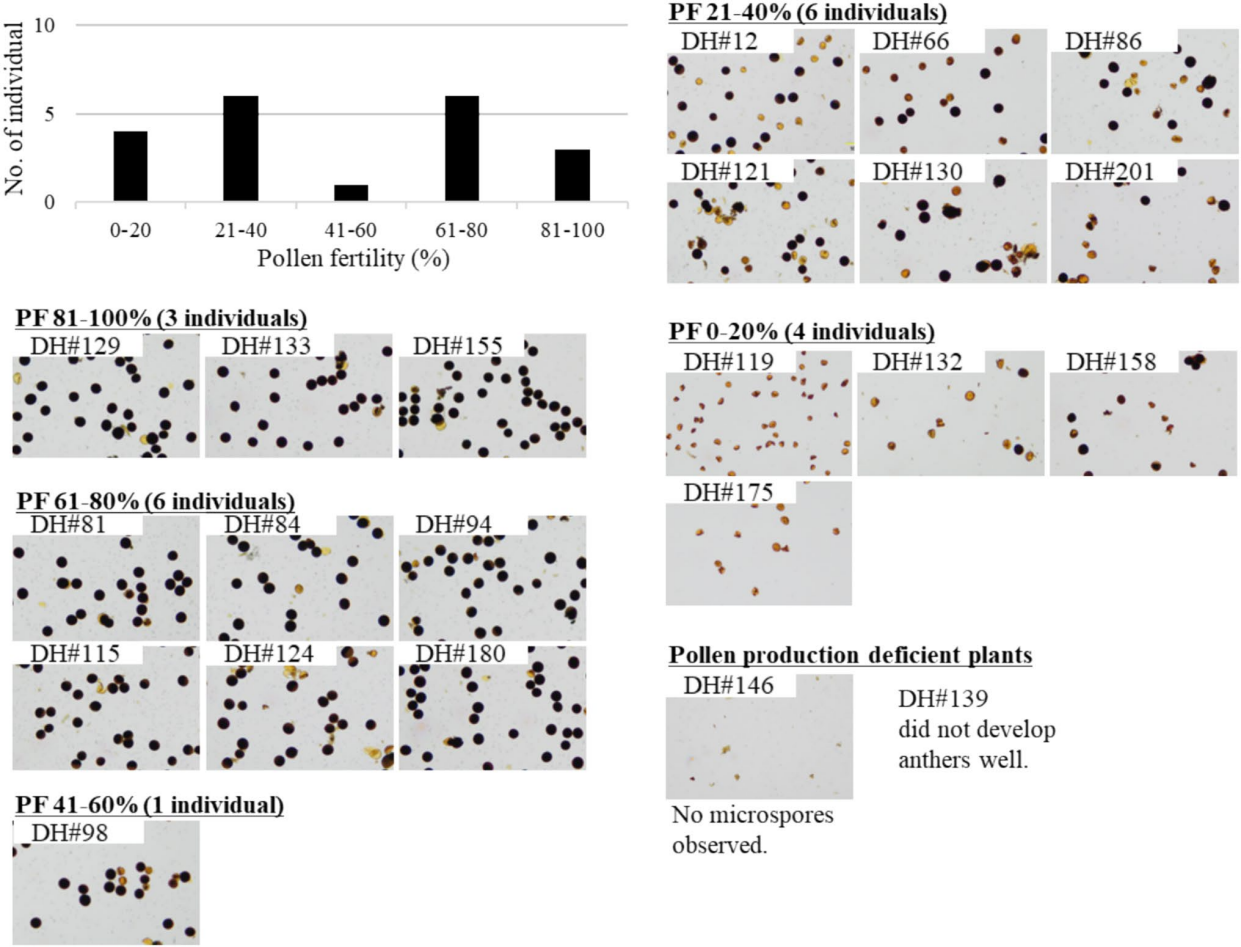
Pollen fertility in the DH_1_ population. Pollen was stained with I_2_-Ki staining. Among 22 diploid DH plants, 9 individuals showed more than 60% pollen fertility. Three DH plants, DH#129, DH#133, and DH#155, showed more than 80% pollen fertility, almost equivalent to common diploid rice. Although all DH plants were genetically fixed, several plants showed severe pollen grain sterility

In the 22 DH_1_ plants, nine individuals showed pollen fertility of more than 60%, and the fertility of three individuals—DH#129, DH#133, and DH#155—was higher than 80%, which was almost equivalent to that of normal diploid rice (Fig. 3, Table 4). Seven individuals showed 21–60% partial pollen fertility in the other DH plants (Fig. 3, Table 4). These fertile and partially fertile DH plants, except for DH#86 and DH#121, set seeds of more than 10 grains by self-pollination, and the seed-setting rates of these plants ranged from 13.6 to 82.4% (Fig. S3, Table 4). These results suggest that interspecific hybrids between *O. sativa* and *O. glaberrima* can be pollen-fertile by fixation of the HS loci.

**Table 4:** Pollen fertility and seed-setting rate by self pollination in the DH_1_ population

Plant material	Generation	Pollen fertility (%)	Seed-setting rate (%)
DH#12	DH_1_	37.7	68.2
DH#66	DH_1_	22.4	29.1
DH#81	DH_1_	69.5	56.1
DH#84	DH_1_	66.0	59.1
DH#86	DH_1_	30.3	13.6
DH#94	DH_1_	71.3	82.4
DH#98	DH_1_	56.1	24.4
DH#115	DH_1_	69.4	73.2
DH#119	DH_1_	0.0	0.0
DH#121	DH_1_	39.4	13.6
DH#124	DH_1_	65.0	16.0
DH#129	DH_1_	84.8	61.5
DH#130	DH_1_	35.4	72.1
DH#132	DH_1_	0.7	0.0
DH#133	DH_1_	80.2	64.7
DH#139	DH_1_	n.d. *^1^	0.0
DH#146	DH_1_	n.d. *^2^	0.0
DH#155	DH_1_	87.5	58.8
DH#158	DH_1_	4.7	0.0
DH#175	DH_1_	0.0	0.0
DH#180	DH_1_	77.9	26.7
DH#201	DH_1_	26.5	19.3

^*1^Anthers did not develop well

^*2^No pollens nor microspores were obesrved within the anthers

Although their HS loci were fixed, four individuals showed PF lower than 20%, especially three individuals (DH#119, DH#132, and DH#175) that were almost sterile as their fertility was 0–0.7% (Fig. 3, Table 4). The remaining two individuals (DH#139 and DH#146) did not produce pollen, and DH#146 did not develop anthers (Fig. 3).

### Genomic composition of diploid DH plants

Whole-genome analysis was performed using GRAS-Di analysis to analyze the genomic composition of plants in the DH_1_ population. Polymorphic regions between PL9-2X and WK21-2X were used to analyze the genomic compositions of the DH plants. The minimum distance between adjacent polymorphic sites was set to 100 kb to accurately analyze the genome of the DH plants. A total of 1,292 polymorphic sites were used for the comprehensive genomic analysis (Table 5).

**Table 5:** Ratio of the *sativa* allele (*S* ratio) within the genome of interspecific hybrid plants in the DH_1_ population

Chr	All	1	2	3	4	5	6	7	8	9	10	11	12
No. of genotyped locus	1292	169	142	147	112	116	107	87	85	81	78	86	82
Average distance between adjacent loci (Mb ± S.D.)	0.29 ± 0.21	0.26 ± 0.18	0.25 ± 0.15	0.24 ± 0.13	0.32 ± 0.25	0.26 ± 0.17	0.29 ± 0.21	0.34 ± 0.26	0.33 ± 0.23	0.28 ± 0.18	0.30 ± 0.20	0.34 ± 0.24	0.34 ± 0.30
PL9	1.00	1.00	1.00	1.00	1.00	1.00	1.00	1.00	1.00	1.00	1.00	1.00	1.00
WK21	0.00	0.00	0.00	0.00	0.00	0.00	0.00	0.00	0.00	0.00	0.00	0.00	0.00
DH#12	0.81	1.00	0.81	0.74	0.71	0.63	0.89	1.00	1.00	0.56	1.00	0.49	0.91
DH#66	0.50	0.82	0.50	0.33	0.98	0.61	0.56	0.08	0.24	1.00	0.09	0.10	0.26
DH#81	0.74	0.77	0.74	0.33	0.96	0.99	0.83	0.90	0.88	0.40	0.92	0.28	0.98
DH#84	0.64	1.00	0.65	0.32	0.23	0.99	0.78	0.22	1.00	0.93	0.53	0.02	0.91
DH#86	0.59	0.86	0.14	0.23	0.66	0.99	0.78	0.83	0.88	0.70	0.76	0.02	0.34
DH#94	0.80	1.00	0.86	0.33	0.95	0.97	0.90	0.72	0.88	0.70	0.92	0.33	0.98
DH#98	0.62	1.00	0.28	0.32	0.61	0.99	0.78	0.90	0.88	0.10	0.00	0.47	0.98
DH#115	0.62	0.53	1.00	0.72	0.58	0.22	0.10	0.17	1.00	1.00	0.35	1.00	0.85
DH#119	0.63	0.82	0.44	0.32	0.96	0.97	0.90	0.40	1.00	0.86	0.00	0.02	0.75
DH#121	0.67	0.77	0.68	0.03	0.96	0.97	0.78	0.71	1.00	0.63	0.55	0.91	0.20
DH#124	0.48	0.55	1.00	0.18	0.64	0.61	0.67	0.17	0.19	0.44	0.00	0.07	0.84
DH#129	0.66	0.77	0.75	0.32	0.74	1.00	0.83	0.24	1.00	0.57	0.00	0.53	0.98
DH#130	0.64	0.82	0.40	0.20	0.86	0.97	0.90	0.83	0.88	0.01	1.00	0.33	0.48
DH#132	0.68	1.00	0.72	0.33	0.96	1.00	0.90	0.21	1.00	0.30	0.00	1.00	0.32
DH#133	0.71	0.93	0.74	0.21	0.95	0.96	0.77	0.31	1.00	0.66	0.48	0.81	0.56
DH#139	0.63	0.90	1.00	0.22	0.84	0.99	0.70	0.27	0.15	0.91	1.00	0.00	0.15
DH#146	0.51	0.89	0.30	0.03	0.75	0.68	0.74	0.90	0.50	0.40	0.74	0.00	0.00
DH#155	0.59	0.96	0.49	0.34	0.81	0.65	0.24	0.91	0.61	1.00	0.61	0.11	0.16
DH#158	0.52	0.95	0.11	0.32	0.72	0.99	0.78	0.85	1.00	0.06	0.00	0.13	0.00
DH#175	0.47	0.82	0.29	0.50	0.74	0.21	0.52	0.92	0.07	0.00	0.34	0.13	0.85
DH#180	0.64	0.85	0.66	0.03	0.96	0.99	0.78	0.27	0.88	0.58	0.92	0.02	0.67
DH#201	0.50	0.76	0.71	0.16	0.63	0.18	0.70	0.39	0.25	0.88	1.00	0.00	0.20
Average	0.62	0.85	0.60	0.30	0.78	0.80	0.72	0.55	0.74	0.58	0.51	0.31	0.57
HS locus on the chromosome		*S58*	*S29(t)*	*S19*	*S2*	–	*S1*	*S20, S21*	–	–	–	–	–

**Fig. 4:**
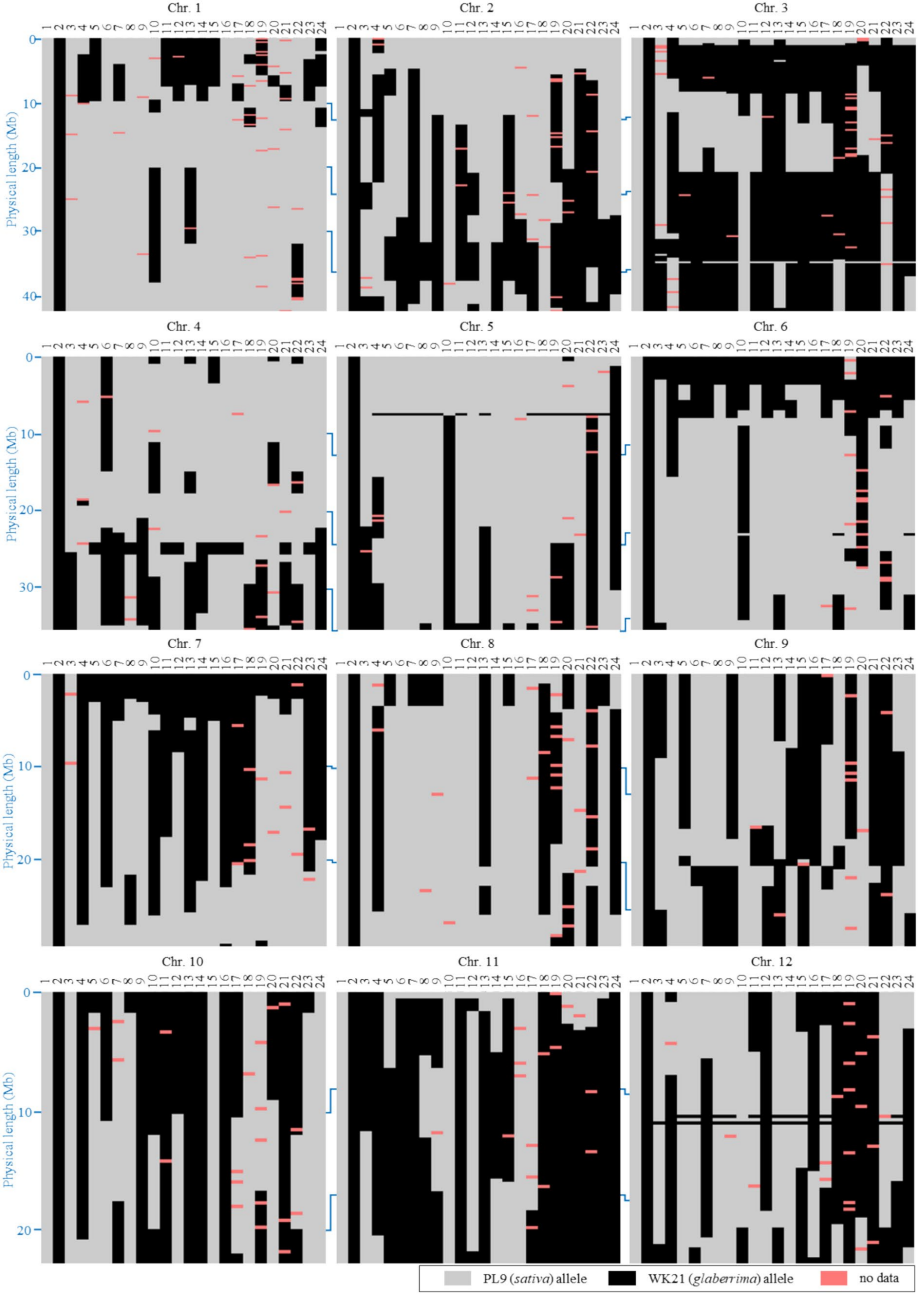
Graphical genotype of the DH_1_ materials. Gray and black regions indicate the genomic regions inherited from PL9-2X and WK21-2X, respectively. 1: PL9-2X, 2: WK21-2X, 3: DH#12, 4: DH#66, 5: DH#81, 6: DH#84, 7: DH#86, 8: DH#94, 9: DH#98, 10: DH#115, 11: DH#119, 12: DH#121, 13: DH#124, 14: DH#129, 15: DH#130, 16: DH#132, 17: DH#133, 18: DH#139, 19: DH#146, 20: DH#155, 21: DH#158, 22: DH#175, 23: DH#180, and 24: DH#201. The graphical genotype images were built using Flapjack software

**Fig. 5:**
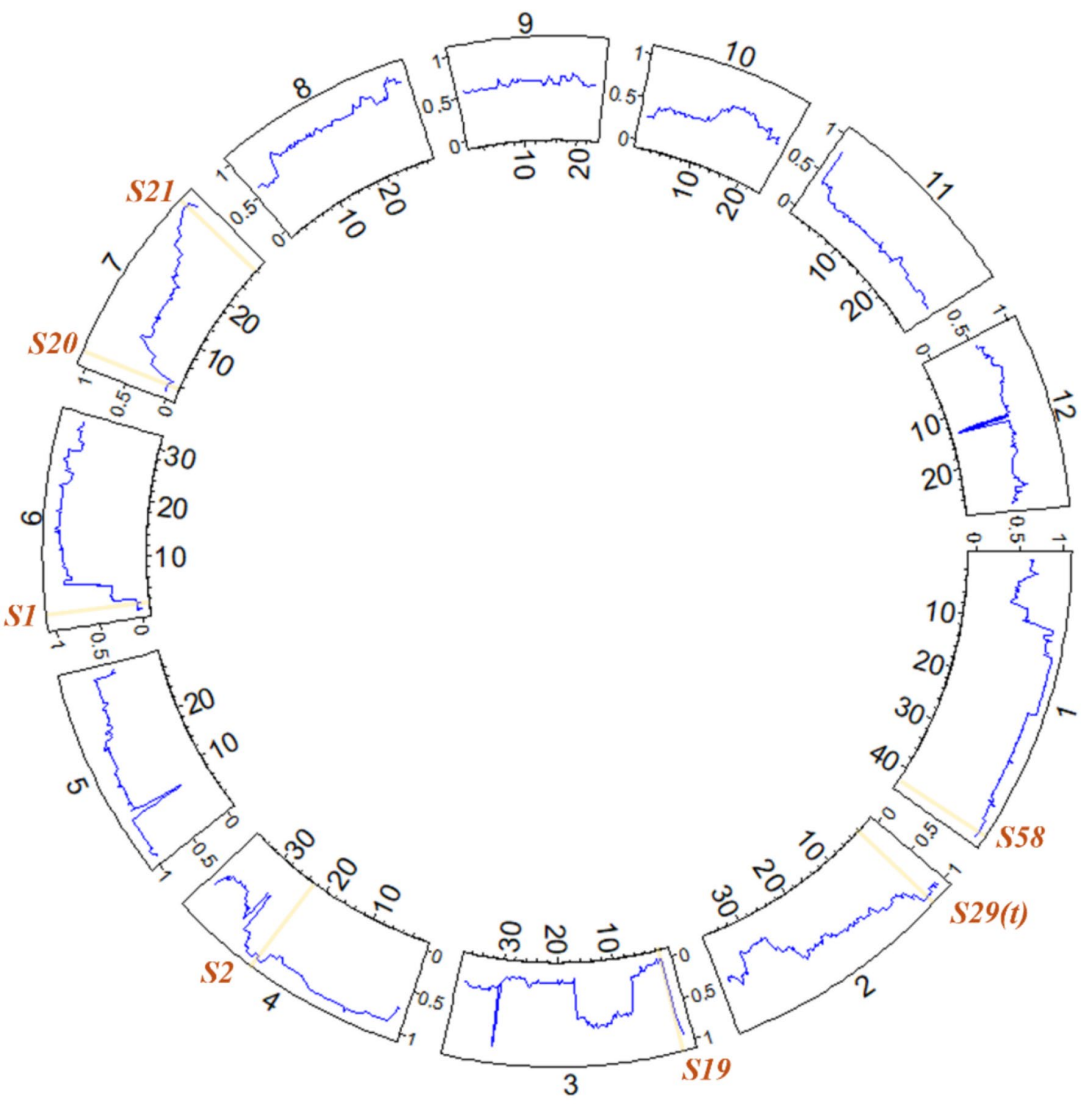
Overview of the *sativa* allele frequency (*S* ratio) across all chromosomes of the DH_1_ population. The numbers 1–12 outside of the circle image indicate chromosome number. The numbers inside the circle and X-axis indicate the physical position (Mb) within each chromosome. The Y-axis indicates the *S* ratio (0–1). The expected *S* ratio in all regions was 0.5 if no factors affected allele transmission during anther culture. The regions where the HS loci are located are shaded with yellow. The circular image was produced using the circlize package for R

The DH plants were confirmed to be genetically balanced between the two parents, PL9-2X and WK21-2X (Fig. 4). The ratio of the *O. sativa* allele (*S* ratio) in each genetic region was evaluated as an indicator of the genomic composition of DH plants (Fig. 5, Table 5). The *S* ratio would be 0.5 if no genetic bias existed in allele frequency toward either of the alleles of the two parental lines, PL9-2X (*O. sativa*) and WK21-2X (*O. glaberrima*). The average *S* ratios for each chromosome of the DH plants are listed in Table 5. The average *S* ratio within each DH plant throughout the entire chromosome ranged from 0.47 in DH#175 to 0.81 in DH#12, and the average *S* ratio for all DH plants was 0.62, which was slightly biased toward the *sativa* allele (Table 5). At the chromosome level, chromosomes 1, 2, 3, and 4 each contained a single HS locus that caused transmission ratio distortion of the HS allele toward either *O. sativa* (*S2*, *S29(t)*, *and S58*) or *O. glaberrima* (*S19*); the *S* ratios of these chromosomes were biased toward one of the parental alleles (Fig. 5, Tables 5 and Table S6). On chromosome 6, where the *S1* locus causing pollen grain sterility is located, the *S* ratio in the region around the *S1* locus was biased toward the *glaberrima* allele, whereas the average *S* ratio of this chromosome was biased toward the *sativa* rather than the *glaberrima* allele (Figs. 4 and 5, Table 5). On chromosome 7, where *S20* caused transmission ratio distortion toward the *glaberrima* and *S21* caused transmission ratio distortion toward the *sativa* alleles located on the short and long arms, respectively, it showed a neutral *S* ratio of 0.55 across the chromosome on average, and the *S* ratio around each HS locus was locally biased toward either of the alleles (Fig. 5, Table 5).

The HS loci in all DH plants were fixed in a homozygous state of the preferentially transmitting allele, except for DH#12 (Figs. 4 and S4, Table S6). All HS loci in DH#12 were fixed in homozygous for the *sativa* allele, even at loci *S1*, *S19*, and *S20,* where the *glaberrima* allele was preferentially transmitted (Fig. S4, Table S6).

In addition, the regions around *HWS1* and *HWS2* were genotyped using additional sets of DNA markers (Tables 6 and Table S4), which were the causal factors of pollen grain sterility between *O. sativa* and *O. glaberrima* in the progeny generations (Liao et al. 2024). Among the DH_1_ plants, three individuals, DH#132, DH#139, and DH#146, lost both functional *HWS* genes, whereas the other DH plants retained functional *HWS1* and/or *HWS2* (Table 6). All three DH plants without functional *HWS* genes were pollen-sterile during the development of pollen (DH#132 and DH#146) or anthers (DH#139) (Fig. 3, Table 4). These results suggest that interspecific hybrids between *O. sativa* and *O. glaberrima* require functional *HWS1* and/or *HWS2* genes for pollen fertility, in addition to the fixation of the HS loci.

**Table 6:** Genotypic analysis of the DH_1_ plants in the regions around the *HWS1* and *HWS2* genes

Plant material	*HWS1* region^*1^				*HWS2* region^*1^			Genotype		Sterility estimation	Pollen fertility (%)
	GRAS-Di site	DNA marker	DNA marker	GRAS-Di site	GRAS-Di site	DNA marker	GRAS-Di site				
	S01_7042943	c1-7.38	c1-7.42	S01_7734020	S12_11557810	c12-11.91	S12_11947277	*HWS1*	*HWS2*		
PL9	*S*	*S*	*S*	*S*	*S*	*S*	*S*	*S*	*S*	–	–
WK21	*G*	*G*	*G*	*G*	*G*	*G*	*G*	*G*	*G*	–	–
DH#12	*S*	*S*	*S*	*S*	*S*	*S*	*S*	*S*	*S*	–	37.7
DH#66	*G*	*G*	*G*	*G*	*G*	*G*	*G*	*G*	*G*	–	22.4
DH#81	*G*	*G*	*G*	*G*	*S*	*S*	*S*	*G*	*S*	–	69.5
DH#84	*S*	*S*	*S*	*S*	*S*	*S*	*S*	*S*	*S*	–	66.0
DH#86	*G*	*G*	*G*	*G*	*G*	*G*	*G*	*G*	*G*	–	30.3
DH#94	*S*	*S*	*S*	*S*	*S*	*S*	*S*	*S*	*S*	–	71.3
DH#98	*S*	*S*	*S*	*S*	*S*	*S*	*S*	*S*	*S*	–	56.1
DH#115	*S*	*S*	*S*	*S*	*S*	*S*	*S*	*S*	*S*	–	69.4
DH#119	*G*	*G*	*G*	*S*	*S*	*S*	*S*	*G*	*S*	–	0.0
DH#121	*G*	*G*	*G*	*G*	*G*	*G*	*G*	*G*	*G*	–	39.4
DH#124	*S*	*S*	*S*	*S*	*S*	*S*	*S*	*S*	*S*	–	65.0
DH#129	*G*	*G*	*G*	*G*	*S*	*S*	*S*	*G*	*S*	–	84.8
DH#130	*G*	*S*	*S*	*S*	*S*	*S*	*S*	*S*	*S*	–	35.4
DH#132	*S*	*S*	*S*	*S*	*G*	*G*	*G*	*S*	*G*	Sterile	0.7
DH#133	*G*	*G*	*G*	*G*	*S*	*S*	*S*	*G*	*S*	–	80.2
DH#139	*S*	*S*	*S*	*S*	-	*G*	*G*	*S*	*G*	Sterile	n.d.^*2^
DH#146	*S*	*S*	*S*	*S*	*G*	*G*	*G*	*S*	*G*	Sterile	n.d.^*3^
DH#155	*G*	*G*	*G*	*S*	*G*	*G*	*G*	*G*	*G*	–	87.5
DH#158	*G*	*G*	*G*	*G*	*G*	*G*	*G*	*G*	*G*	–	4.7
DH#175	*S*	*S*	*S*	*S*	*S*	*S*	*S*	*S*	*S*	–	0.0
DH#180	*G*	*G*	*G*	*G*	*S*	*S*	*S*	*G*	*S*	–	77.9
DH#201	*G*	*G*	*G*	*S*	*G*	*G*	*G*	*G*	*G*	–	26.5

^*1^Non-functional alleles for the *HWS* genes are represented by gray shading

^*2^Anthers did not develop well

^*3^No pollens nor microspores were observed within anthers

### Agronomic traits of DH plants

Ten individuals from the DH_1_ population successfully developed the next population (the DH_2_ population), and the agronomic traits of these 10 DH lines were evaluated. The field trial data provide useful preliminary insights; however, the limitation of sample size and the lack of replicates should be acknowledged. Therefore, these results should be interpreted with some caution.

**Fig. 6:**
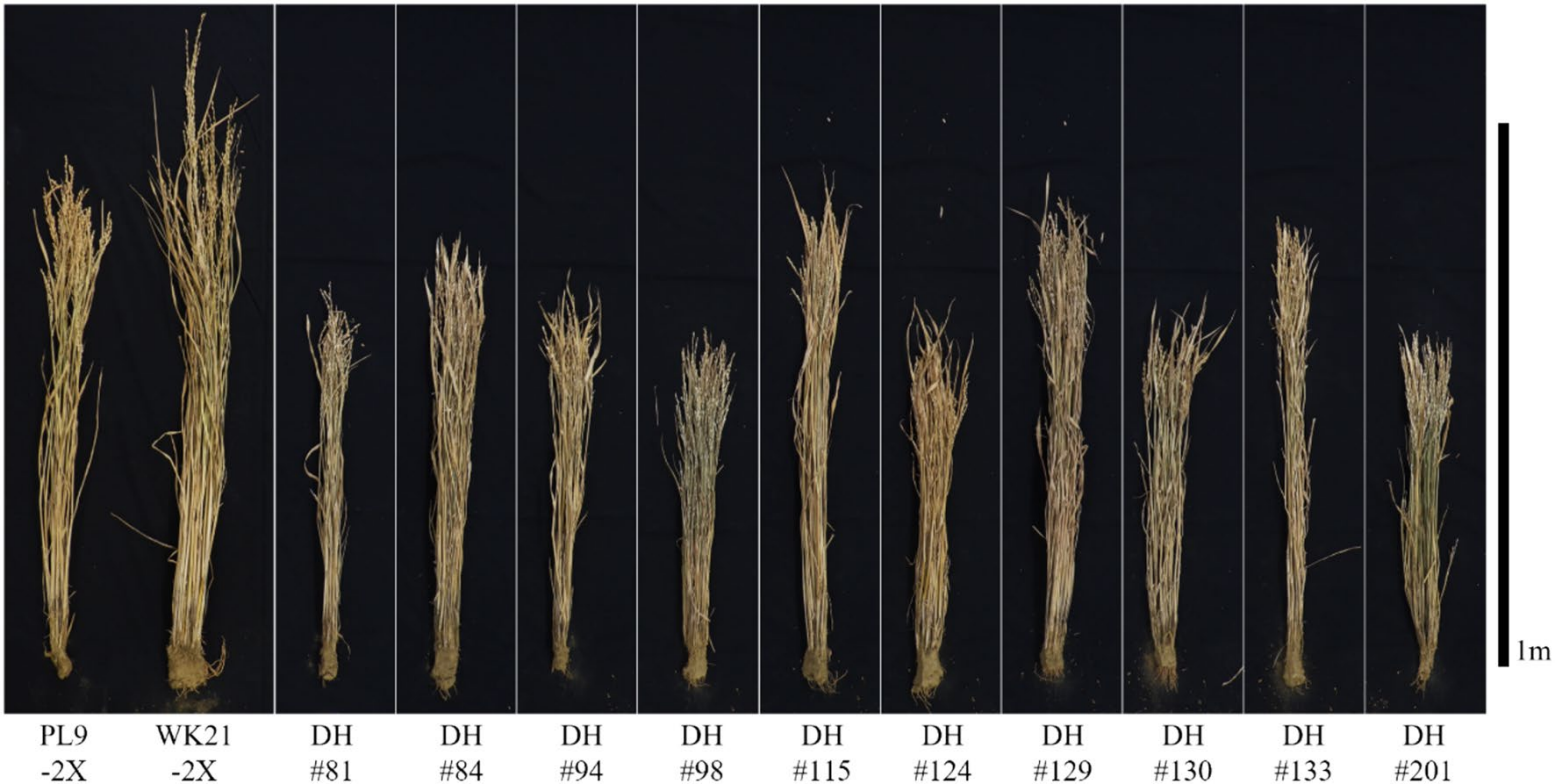
DH_2_ lines and parental lines of the DH, PL9-2X, and WK21-2X

Among a total of nine traits, seven (CL, PL, PW, HI, TS, TGW, and DTH) exhibited variation among the DH lines (*p* < 0.05, Table S7). CL and PL in the DH lines ranged from 43.5 to 73.7 cm and 14.5 to 24.4 cm, respectively, and tended to be shorter than those in the parental lines PL9-2X and WK21-2X (Figs. 6 and S5, Table 7). HI, which indicates the ratio of PW to the total weight of the plant (PW + CW), ranged from 0.24 to 0.61 in the DH lines, and HI values of three DH lines (DH#94, DH#130, and DH#133) were notably higher than those in the parental line WK21-2X (*p* < 0.05, Table 7). The number of TS was lowest in DH#81 at 49.0 and highest in DH#129 at 127.3, showing a difference of approximately 2.6-fold (Table 7). Seed size varied markedly among the DH_1_ individuals, and TGW in the DH_2_ generation ranged from 17.0 to 35.3 g (Fig. S3, Table 7). DTH ranged between 68.7 and 82.7 days, and DH#84 (78.0), DH#115 (79.3), and DH#129 (82.7) headed notably later than PL9-2X (66.3) but were not significantly different from the WK21-2X parent (72.7) (*p* < 0.05, Table 7). Although the number of plants in this evaluation was limited, requiring further detailed evaluation of agronomic traits in each line, these results suggest that the DH materials exhibit genetic and phenotypic variation as interspecific balanced hybrids between the two species. The DH lines consistently exhibited PF and SR in the DH_2_ generation (Table S8).

**Table 7:** Phenotypic evaluation of parental lines PL9-2X and WK21-2X, and DH materials in the DH_2_ population

Plant material	CL (cm)		PL (cm)		PN		CW (g)		PW (g)		HI (%)		TS		TGW (g)		DTH (days)	
PL9-2X	70.6 ± 2.2	bcd	20.4 ± 0.7	ab	16.0 ± 0.8	a	17.1 ± 0.9	abcd	29.6 ± 3.3	c	0.63 ± 0.01	e	111.3 ± 14.4	cd	28.6 ± 0.8	abc	66.3 ± 2.1	a
WK21-2X	88.4 ± 7.5	d	34.3 ± 2.4	b	11.7 ± 4.5	a	48.9 ± 7.9	cd	18.8 ± 2.8	abc	0.28 ± 0.04	ab	120.0 ± 24.1	d	25.4 ± 3.4	abc	72.7 ± 2.5	abc
DH#81	54.3 ± 1.8	abc	17.1 ± 1.6	a	16.3 ± 1.7	a	16.1 ± 1.1	ab	14.1 ± 1.3	ab	0.47 ± 0.01	abcde	49.0 ± 0.8	a	33.5 ± 2.7	bc	70.3 ± 2.1	ab
DH#84	63.8 ± 3.7	abcd	19.9 ± 1.2	ab	21.3 ± 2.4	a	25.2 ± 4.0	abcd	20.6 ± 4.2	abc	0.45 ± 0.02	abcd	60.3 ± 3.3	abc	32.2 ± 1.7	bc	78.0 ± 1.6	bc
DH#94	60.0 ± 0.6	abcd	15.3 ± 1.5	a	20.0 ± 2.2	a	14.9 ± 2.5	a	23.1 ± 2.4	abc	0.61 ± 0.02	de	73.0 ± 5.7	dbca	35.3 ± 0.9	c	69.3 ± 3.4	ab
DH#98	53.1 ± 2.0	ab	16.3 ± 1.8	a	18.7 ± 3.1	a	18.9 ± 3.0	abcd	5.9 ± 1.3	a	0.24 ± 0.02	a	70.7 ± 5.3	abcd	26.4 ± 4.0	abc	68.7 ± 3.9	ab
DH#115	70.5 ± 2.6	bcd	21.0 ± 0.9	ab	20.7 ± 2.4	a	22.8 ± 3.0	abcd	27.2 ± 2.9	bc	0.55 ± 0.01	bcde	122.3 ± 11.0	d	23.1 ± 1.2	ab	79.3 ± 1.2	bc
DH#124	43.5 ± 0.2	a	14.5 ± 0.3	a	29.7 ± 9.5	a	18.6 ± 4.7	abcd	20.2 ± 3.2	abc	0.52 ± 0.03	abcde	107.3 ± 4.5	bcd	17.0 ± 1.0	a	76.0 ± 3.7	abc
DH#129	73.7 ± 1.0	cd	24.4 ± 0.6	b	24.7 ± 3.9	a	37.6 ± 6.0	bd	38.1 ± 6.2	c	0.50 ± 0.00	abcde	127.3 ± 5.4	d	25.5 ± 1.0	abc	82.7 ± 1.2	c
DH#130	53.9 ± 1.5	abc	18.5 ± 1.7	ab	19.7 ± 3.7	a	18.6 ± 3.6	abcd	25.4 ± 5.6	abc	0.58 ± 0.06	cde	58.3 ± 3.1	ab	34.2 ± 1.0	c	73.7 ± 2.1	abc
DH#133	73.1 ± 1.8	bcd	19.8 ± 1.0	ab	16.7 ± 1.2	a	14.9 ± 0.9	ab	23.7 ± 1.2	abc	0.61 ± 0.01	de	61.0 ± 4.3	abc	29.2 ± 1.8	abc	76.3 ± 2.1	abc
DH#201	53.2 ± 2.4	abc	19.8 ± 0.9	ab	18.3 ± 5.3	a	18.5 ± 4.5	abcd	11.8 ± 2.5	a	0.39 ± 0.02	abc	82.0 ± 9.9	dbca	27.3 ± 2.3	abc	69.0 ± 3.7	ab
Min–Max	43.5–73.7		14.5–24.4		16.3–29.7		14.9–37.6		5.9–38.1		0.24–0.61		49.0–127.3		17.0–35.3		68.7–82.7	

## Discussion

### Tetraploidization and diploid induction facilitate the development of genetically balanced interspecific hybrids

Interspecific hybrids between *O. sativa* and *O. glaberrima* exhibit severe sterility of pollen grains, but they may become pollen-fertile by genetic fixation of all HS loci. In the present study, DH lines with balanced genomes between the two species were obtained through successive anther cultures of tetraploid interspecific hybrids (Figs. 1 and 5, Table 5). However, studies on the development of genetically balanced hybrid lines remain limited.

**Fig. 7:**
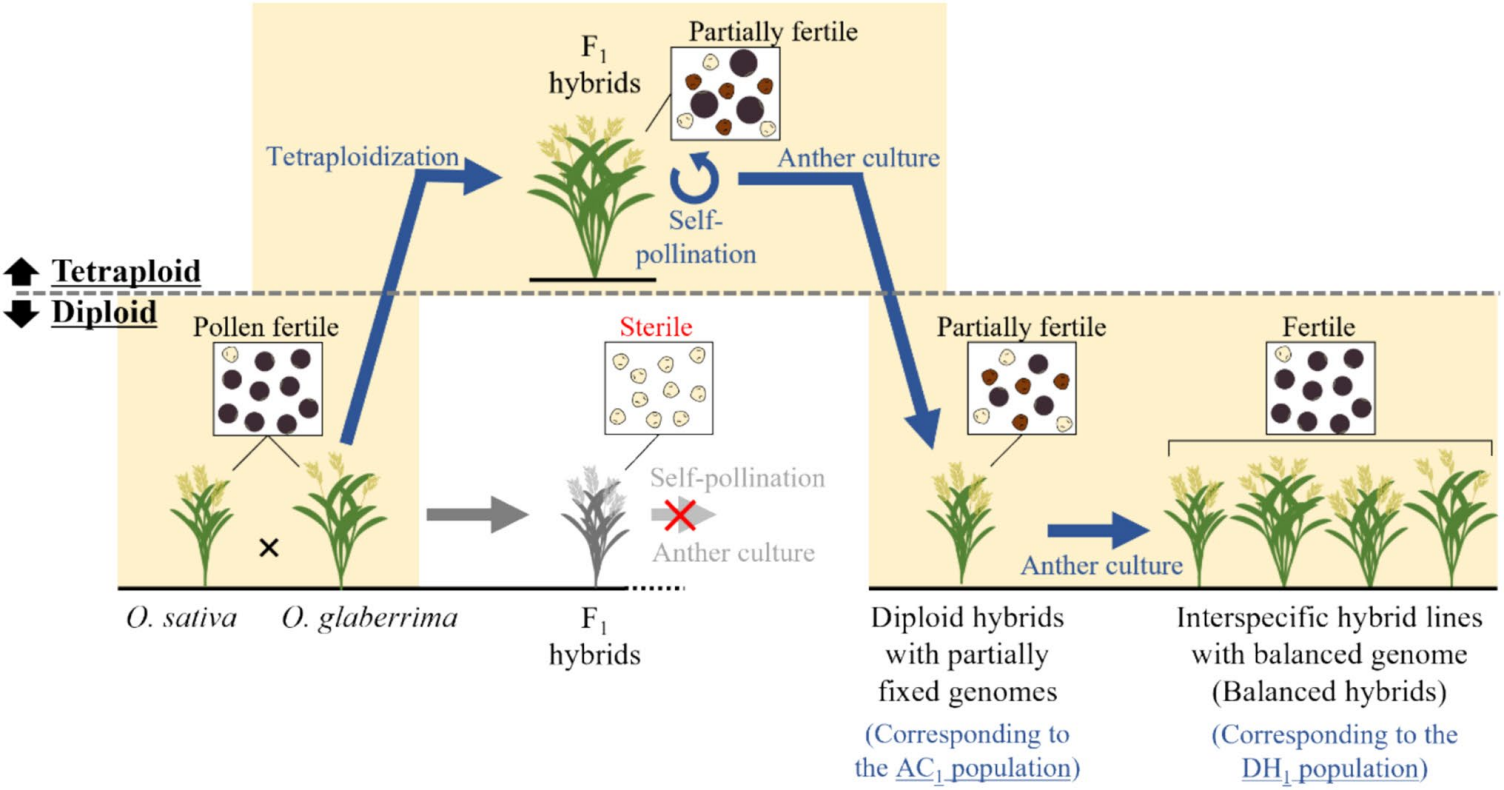
Process for development of the doubled haploid lines with balanced genomes (balanced hybrids) between two species. Diploid interspecific F_1_ hybrids between the two species are pollen-sterile owing to the accumulation of multiple HS loci; thus, the balanced hybrids, such as recombinant inbred lines or DH lines, are difficult to obtain from diploid F_1_ hybrids through self-pollination or anther culture. The key point for producing fertile, balanced hybrids is the fixation of the HS loci to suppress its sterility effect. The present study shows that the fertile hybrid lines with balanced genomes can be produced through tetraploidization to overcome the HS loci, followed by ploidy reduction from tetraploid to diploid and fixation of the HS locus through two rounds of anther culture

Previously, an attempt was made to obtain DH plants through anther culture of the diploid interspecific F_1_ hybrids between *O. sativa* and *O. glaberrima* (Kanaoka et al. 2018; Kuniyoshi et al. 2020), but this was unsuccessful because of severe pollen grain sterility caused by the accumulation of multiple HS loci in the F_1_ generation. It was assumed that diploid interspecific hybrids could become pollen-fertile by fixing part of the multiple HS loci in a homozygous state, and such diploid hybrid plants could successfully produce DH plants through anther culture (Fig. 7). However, a method for developing partially fixed diploid hybrids could not be established without tetraploidization.

In a previous study, it was confirmed that pollen grain sterility caused by the HS locus with the KP system could be mitigated by tetraploidization (Fig. S1) and the AC_1_ plants with a partially fixed genome were produced through anther culture of the WK21-4X/PL9-4X F_2_ hybrids (Figs. 1 and 7) (Kuniyoshi et al. 2024). In the present study, that AC_1_ plants were confirmed that they could partially restore pollen fertility, and the callus induction frequency of AC_1_ plants increased as pollen fertility improved (Fig. 2c). These AC_1_ plants showed a higher frequency of callus formation than the diploid F_1_ hybrids (Table 2), resulting in the production of 22 DH plants in the DH_1_ population with balanced genomes between *O. sativa* and *O. glaberrima* (Table 5). Therefore, overcoming hybrid sterility by tetraploidization and the induction of partially fixed diploid hybrids in the AC_1_ population through anther culture of the tetraploid hybrids are the key steps for the production of DH plants with balanced genomes (Figs. 7 and S1).

### DH plants with balanced genomes of *O. sativa *and *O. glaberrima* could be pollen-fertile

All DH_1_ plants were genetically fixed (Fig. 4) such that all HS loci were homozygous for either the *sativa* or *glaberrima* allele (Fig. S4). Among the DH_1_ plants, nine individuals showed high pollen fertility; ˃ 60% (Fig. 3). Thus, even interspecific hybrids can produce fertile pollen grains if the HS loci become homozygous. The homozygous state of the HS loci was successfully created by tetraploidization, followed by diploid induction, which led to the suppression of the HS loci (Fig. 7). HS loci are major sterility factors in combinations of *O. sativa* and other related species, such as *O. longistaminata*, *O. glumaepatula*, and *O. meridionalis* (Hu et al. 2006; Li et al. 2018; Yu et al. 2018; You et al. 2023; Myint et al. 2024). Genetically balanced fertile hybrids can be produced for these combinations by fixing HS loci.

Although the HS loci were fixed completely, several DH plants still exhibited severe sterility of pollen grains (Fig. 3, Table 4). The *HWS* genes represent another genetic element involved in hybrid sterility between *O. sativa* and *O. glaberrima*, differing from the HS loci. Liao et al. (2024) reported two *HWS* genes within the *Oryza* genome, *HWS1* on chromosome 1 and *HWS2* on chromosome 12, and that functional *HWS1* and *HWS2* genes are only found in *O. glaberrima* and *O. sativa*, respectively. The functional *HWS* gene is essential for anther and pollen development; otherwise, the interspecific hybrids will be pollen-sterile if they lose both functional *HWS* genes (Liao et al. 2024). In the DH_1_ population, three individuals (DH#132, DH#139, and DH#146) were confirmed to have lost both functional *HWS1* and *HWS2* (Table 6), and the pollen of these plants was not available because of severe pollen grain sterility (DH#132), deficiency in pollen (DH#146), or anther (DH#139) development (Fig. 3). Therefore, in addition to the fixation of the HS loci, the presence of the functional *HWS* gene is another essential factor for pollen fertility in interspecific hybrids between *O. sativa* and *O. glaberrima*. DH#119 and DH#175 also showed severe pollen grain sterility, although they retained the functional *HWS* gene(s) (Table 6), suggesting that there is other genetic element that causes hybrid sterility between *O. sativa* and *O. glaberrima*.

### The potential of DH plants

The DH plants varied genetically and phenotypically (Figs. 5, 6, and S3, Table 7). The *S* ratio throughout all chromosomes ranged from 0.47 to 0.81, with an average of 0.62, which was slightly biased toward the *sativa* allele (Table 5). Among the seven HS loci, four (*S2*, *S21*, *S29(t)*, and *S58*) distorted the transmission ratio of the HS allele toward the *O. sativa* allele, whereas the remaining three loci (*S1*, *S19*, and *S20*) resulted in the distortion of the *O. glaberrima* allele. This difference in the number of HS loci in which the allele was preferentially transmitted may be one of the reasons for this overall bias.

Ten of the DH plants produced a subsequent DH_2_ generation (Fig. 6). These 10 lines exhibited variation in the seven traits (CL, PL, PW, HI, TS, TGW, and DTH) (Table S7). Three of the 10 lines showed a higher HI than that of the *O. glaberrima* parental line, WK21-2X. These results indicate that the DH lines may serve as genetically balanced progenies with potential to integrate characteristics of both species (Fig. 1). *O. glaberrima* has conventionally been used to improve the qualitative traits of *O. sativa* varieties through the development of CSSLs by successive backcrossing, using *O. sativa* as the recurrent parent (Fig. 1) (Ali et al. 2010; Bimpong et al. 2011; Garavito et al. 2010; Yamagata et al. 2019). The upland NERICA series as the CSSL varieties have achieved considerable success in sub-Saharan African countries (Somado et al. 2008; Futakuchi et al. 2021), but some of the useful traits of CG14 (*O. glaberrima* parent of NERICA), for example, weed competitive ability and adaptability to low fertile soils, which might be controlled by multiple genetic elements, appeared not to be inherited by the NERICA series (Saito et al. 2012). Yamamoto et al. (2018) analyzed the whole genomic composition of the upland NERICA series (NERICA 1–9) by re-sequencing analysis and revealed that the NERICA series retained the CG14 genome at a low ratio ranging from 1.3 to 9.8% of the total genome with the genetic background of WAB56-104 (Yamamoto et al. 2018). This low ratio of *O. glaberrima* segments in the NERICA could be one of the reasons why the varieties could not inherit the complex traits of *O. glaberrima*.

In the present study, the genomic composition of the DH plants was analyzed based on 1,292 polymorphic sites positioned on average every 0.29 ± 0.21 Mb across all 12 chromosomes, confirming that these materials inherited between 19 and 53% of the *O. glaberrima* genome (Table 5). Although GRAS-Di genotyping is not as precise as re-sequencing, it sufficiently reflects the overall genomic composition of the DH materials given the density of the genotyped sites (Table 5). Thus, the DH materials developed retain higher ratios of the *O. glaberrima* genome than those retained by traditional CSSL varieties. These DH plants are phenotypically diverse (Fig. 6, Table 7), and considering these results, our approach using balanced hybrids provides an alternative to the development of CSSLs, facilitating the analysis of genetic elements that control complex traits in *O. glaberrima* and their utilization in breeding of *O. sativa* varieties.

In addition to the direct use of the DH materials, once promising traits are identified, these DH lines could serve as breeding material for *O. sativa* varieties. The DH materials in the present study retained three HS loci; the *S1*, *S19*, and *S20* loci in the *glaberrima* allele, except for DH#12 (Fig. S4). Li et al. (2023) reported pollen fertility observations in cases where multiple HS loci were accumulated. Their study indicated that when an *O. sativa* NIL carrying three HS loci, the *S1* and *S20* loci (as examined in this study) and the *S37(t)* locus, in the *glaberrima* alleles was crossed with *O. sativa*, the resulting pollen fertility was 3.35%. In the present study, the pollen grain fertility of the diploid hybrids in the AC_1_ population, which carried three HS loci in a heterozygous state, averaged 8.9% (Fig. 2b). These results suggest that hybrids between the DH materials and *O. sativa* will be partially pollen-fertile; therefore, the DH materials could be used for breeding of *O. sativa* through anther culture (Fig. 2c). However, the number of DH lines was limited, and the phenotype evaluation remained preliminary. To evaluate the usability of DH as balanced hybrids, further production of DH and a detailed evaluation of agronomic traits will be necessary. Furthermore, assessing the crossability between DH and *O. sativa* varieties will be pertinent to evaluate them as breeding material for *O. sativa* in the future.

## Supplementary Information

Below is the link to the electronic supplementary material.

Supplementary file1 (DOCX 2004 KB)

## Data Availability

Data sets analyzed in the present report are available in the online supplementary materials. Further information for each experiment is available from the corresponding authors by reasonable requests.
