# Supplementary material for: Fertile interspecific diploid hybrids between the Asian and African rice species facilitated by tetraploidization and its reduction
Source: Theor Appl Genet. 2025 Jun 27;138(7):161. doi: 10.1007/s00122-025-04901-3 (PMC12204913; doi:10.1007/s00122-025-04901-3)
Supplement: Supplementary file 1 — Supplementary file1 (DOCX 2004 KB) [file 122_2025_4901_MOESM1_ESM.docx]

**Fertile interspecific diploid hybrids between the Asian and African rice species facilitated by tetraploidization and its reduction**

**Journal: Theoretical and Applied Genetics**

Kuniyoshi Daichi^1^, Kishima Yuji^2^

^1^ Tropical Agriculture Research Front, Japan International Research Center for Agricultural Sciences, Ishigaki 305-8686, Japan

^2^ Laboratory of Plant Breeding, Research Faculty of Agriculture, Hokkaido University, Sapporo 060-0808, Japan

**Corresponding author**

Kuniyoshi Daichi, Email: kuniyoshid0353@jircs.go.jp


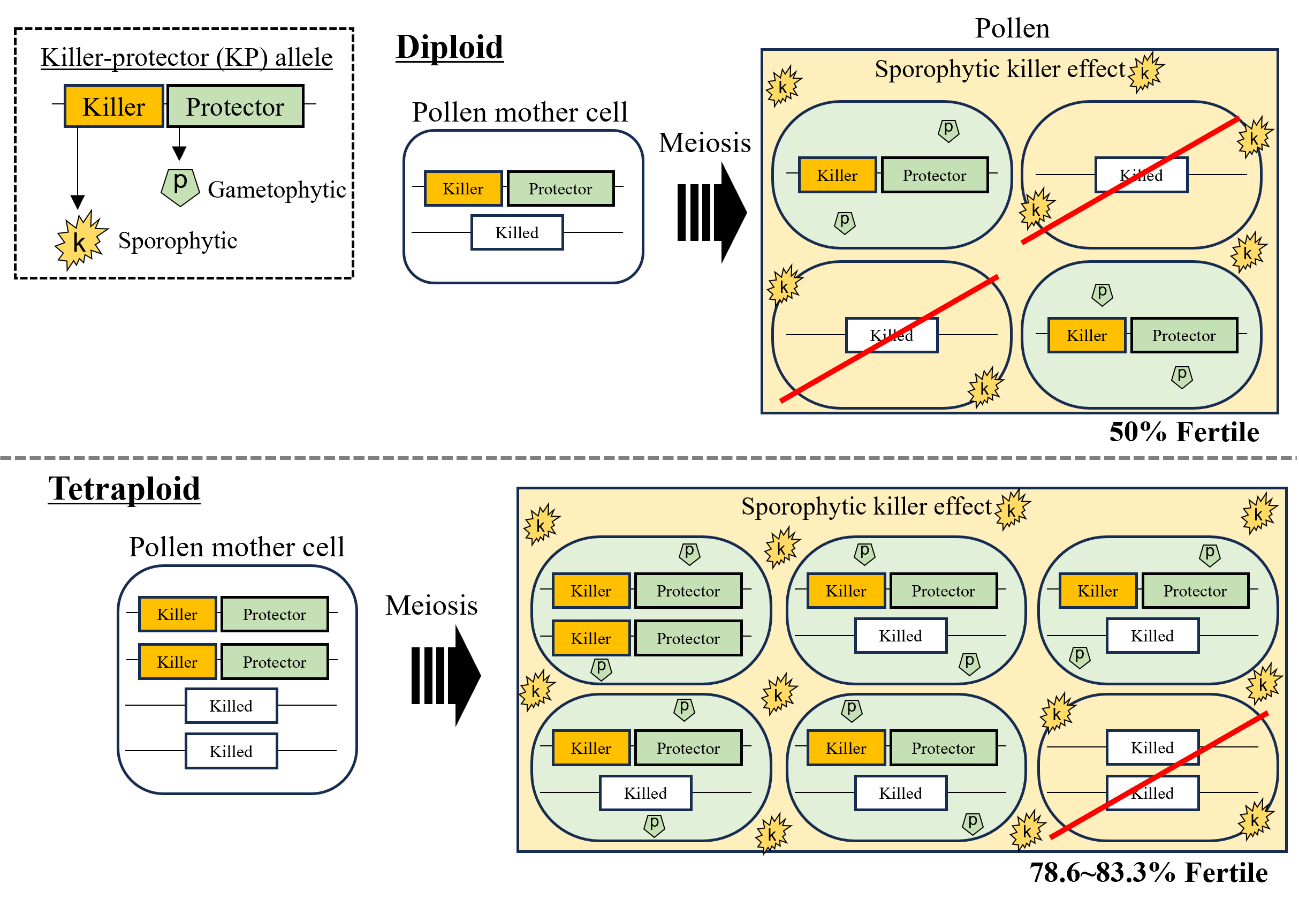
Online figure S1.

Model of Killer-protector mechanism. This model is explained by the interaction of the killer and protector factor. The killer factor induces sterility of pollen in a sporophytic manner, and the protector factor protects the gametes from the effect of the killer factor in a gametophytic manner. The killer and protector tightly linked to each other (referred as to the killer-protector (KP) allele). In diploid plants having a heterozygous HS locus of the KP allele and non-KP allele (referred as to killed allele), half of the pollen carrying the KP allele only survive while other pollen carrying the killed allele will be sterile. While in tetraploid plants, the proportion of the pollen carrying the KP allele increases from 50% to 78.6~83.3% (this percentage depends on the manner of the paring and segregation of homologous chromosomes) and these gametes will survive. Therefore, the sterility effect of the HS locus with the KP mechanism should be reduced in tetraploid conditions.


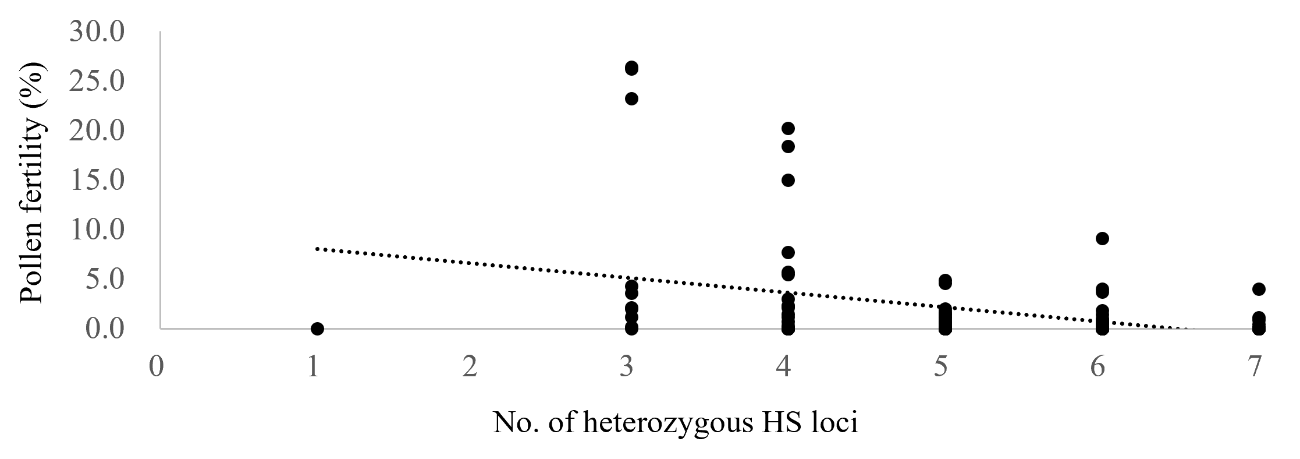


Online Figure S2.

Supplementary Figure S2. Relationship between pollen fertility and number of heterozygous HS loci in the diploid interspecific hybrids of the AC1 population. A weak negative correlation (R=-0.37) was observed between the two elements. The data of the No. of heterozygous HS loci were sourced from Kuniyoshi et al. 2024.


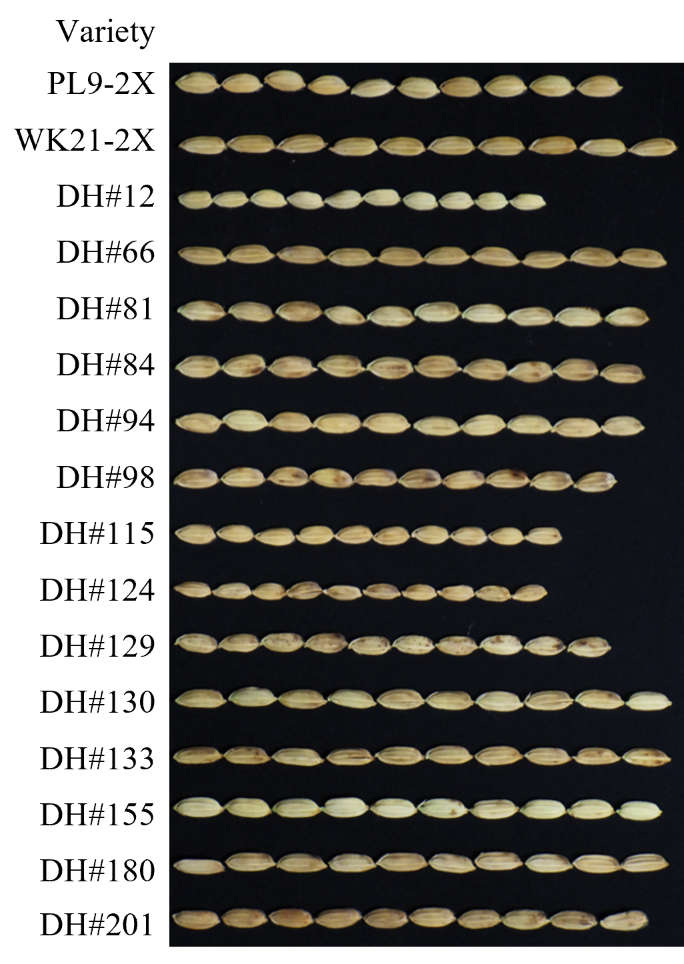


Online figure S3.

Self-pollinated seeds of the DH_1_ individuals and the parental lines of PL9-2X and WK21-2X.


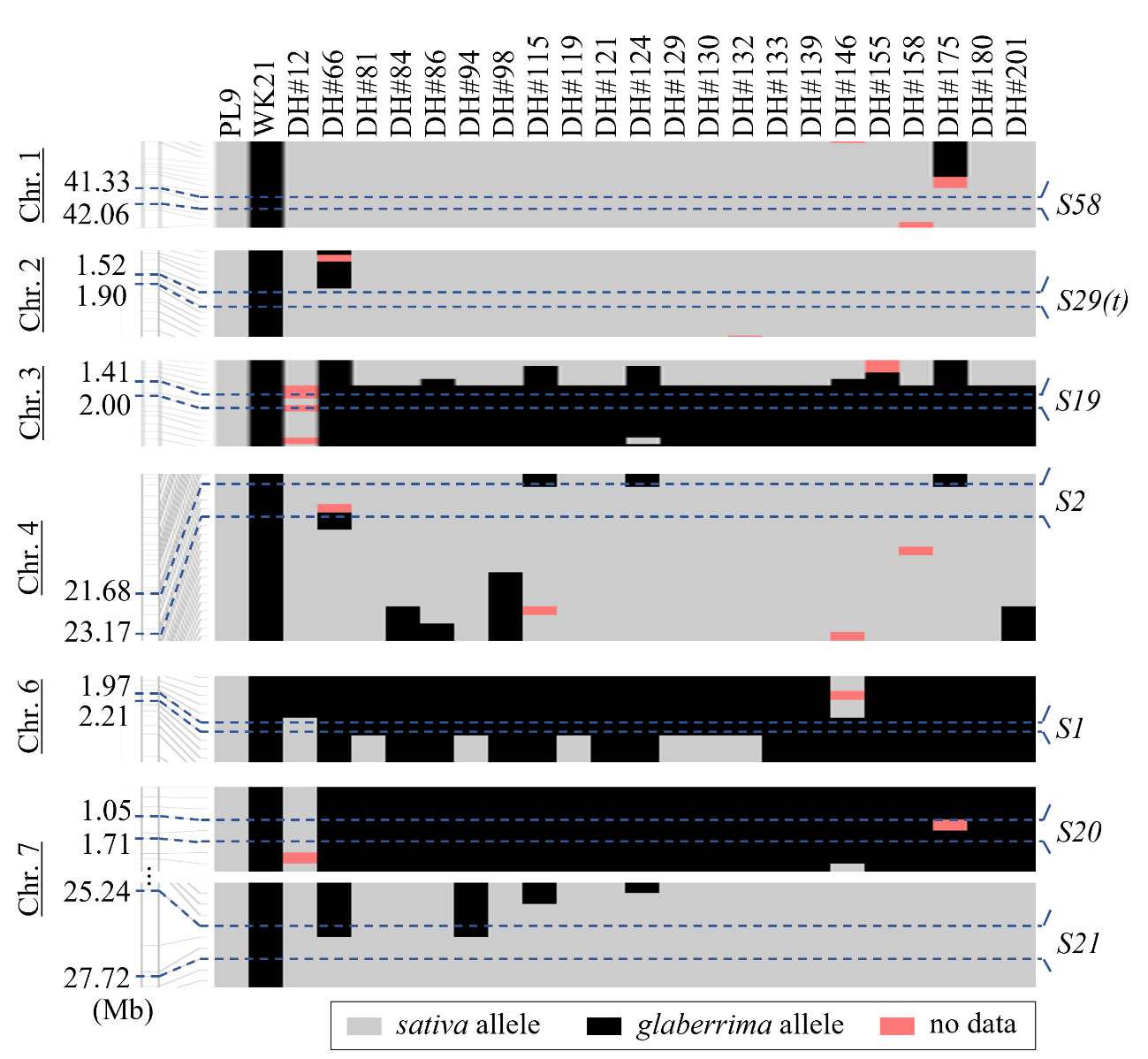


Online figure S4.

Detailed graphical genotypes in the 7 HS regions (S1, S2, S19, S20, S21, S29(t), and S58) of the DH_1_ plants.


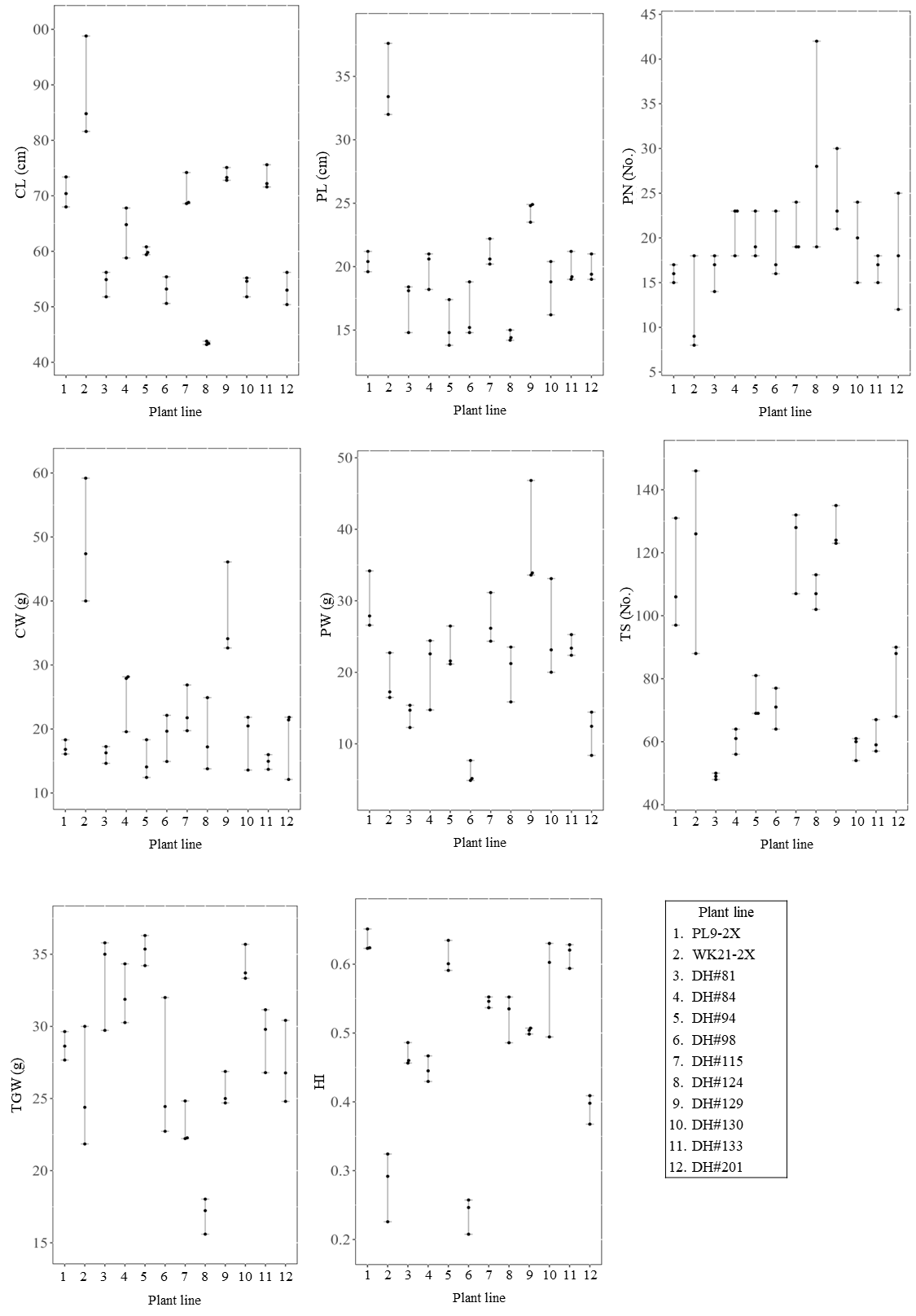


Online figure S5.

Detailed data of evaluation for agronomic traits of the parental varieties and DH_2_ individuals.

# Online table

| Table S1. Pollen fertility and genotypes of the HS loci of the plants in the AC1 population. The data of No. of heterozygous HS were sourced from Kuniyoshi et al. 2024. | | | | | | | | |
| --- | --- | --- | --- | --- | --- | --- | --- | --- |
| Plant ID | No. of  hetero-zygous  HS^*^ | Pollen  fertility (%) | No. of  cultured anthers | No. of anthers  producing calli | Culls induction  frequency % | No. of  replanted calli | No. of  regenerated calli | Regeneration  frequency (%) |
| AC#1 | 5 | 0.0 | 167 | 0 | 0.0 | - | - | - |
| AC#2 | 5 | 0.0 | 137 | 0 | 0.0 | - | - | - |
| AC#3 | 6 | 0.4 | 71 | 0 | 0.0 | - | - | - |
| AC#4 | 3 | 1.2 | 555 | 11 | 2.0 | 11 | 5 | 45.5 |
| AC#5 | 5 | 4.6 | - | - | - | - | - | - |
| AC#6 | 6 | 3.7 | 333 | 0 | 0.0 | - | - | - |
| AC#7 | 3 | 26.2 | 792 | 76 | 9.6 | 64 | 7 | 10.9 |
| AC#8 | 5 | - | 523 | 16 | 3.1 | 16 | 1 | 6.3 |
| AC#9 | 7 | 0.0 | 263 | 0 | 0.0 | - | - | - |
| AC#11 | 6 | 0.0 | 144 | 1 | 0.7 | 1 | 0 | 0.0 |
| AC#12 | 4 | 0.0 | - | - | - | - | - | - |
| AC#13 | 4 | 5.7 | 84 | 0 | 0.0 | - | - | - |
| AC#14 | 6 | 0.6 | 144 | 1 | 0.7 | 1 | 0 | 0.0 |
| AC#15 | 4 | 18.4 | 114 | 3 | 2.6 | 3 | 1 | 33.3 |
| AC#16 | 6 | 0.0 | 289 | 0 | 0.0 | - | - | - |
| AC#17 | 6 | 0.0 | 240 | 0 | 0.0 | - | - | - |
| AC#18 | 5 | 0.0 | 120 | 0 | 0.0 | - | - | - |
| AC#19 | 5 | 2.0 | - | - | - | - | - | - |
| AC#20 | 5 | 0.0 | - | - | - | - | - | - |
| AC#21 | 4 | 1.2 | 66 | 0 | 0.0 | - | - | - |
| AC#22 | 5 | 1.5 | 130 | 0 | 0.0 | - | - | - |
| AC#23 | 5 | 1.7 | 184 | 0 | 0.0 | - | - | - |
| AC#24 | 4 | 0.0 | - | - | - | - | - | - |
| AC#25 | 6 | 1.0 | 969 | 8 | 0.8 | 6 | 0 | 0.0 |
| AC#26 | 3 | 0.0 | - | - | - | - | - | - |
| AC#27 | 5 | 0.0 | - | - | - | - | - | - |
| AC#28 | 4 | 0.0 | 348 | 0 | 0.0 | - | - | - |
| AC#30 | 6 | 0.0 | - | - | - | - | - | - |
| AC#32 | 5 | 0.0 | 323 | 1 | 0.3 | 1 | 0 | 0.0 |
| AC#33 | 7 | 0.0 | 1122 | 11 | 1.0 | - | - | - |
| AC#34 | 5 | - | 552 | 4 | 0.7 | - | - | - |
| AC#35 | 3 | - | - | - | - | - | - | - |
| AC#37 | 4 | 2.3 | 48 | 0 | 0.0 | - | - | - |
| AC#38 | 7 | 0.0 | 143 | 0 | 0.0 | - | - | - |
| AC#39 | 5 | 0.3 | 399 | 0 | 0.0 | - | - | - |
| AC#40 | 4 | 0.0 | 208 | 0 | 0.0 | - | - | - |
| AC#41 | 6 | 0.0 | 951 | 1 | 0.1 | 1 | 1 | 100.0 |
| AC#42 | 7 | - | - | - | - | - | - | - |
| AC#43 | 6 | 1.4 | 60 | 0 | 0.0 | - | - | - |
| AC#44 | 6 | 0.0 | 168 | 0 | 0.0 | - | - | - |
| AC#45 | 6 | 0.6 | - | - | - | - | - | - |
| AC#46 | 3 | 2.1 | 108 | 5 | 4.6 | 5 | 0 | 0.0 |
| AC#47 | 5 | - | 288 | 7 | 2.4 | 7 | 6 | 85.7 |
| AC#48 | 4 | 0.0 | 72 | 0 | 0.0 | - | - | - |
| AC#49 | 4 | - | - | - | - | - | - | - |
| AC#50 | 5 | 0.6 | 309 | 0 | 0.0 | - | - | - |
| AC#51 | 3 | 4.3 | 246 | 3 | 1.2 | 3 | 2 | 66.7 |
| AC#52 | 5 | 0.0 | 316 | 0 | 0.0 | - | - | - |
| AC#53 | 4 | - | 144 | 0 | 0.0 | - | - | - |
| AC#54 | 7 | 0.0 | 978 | 2 | 0.2 | 2 | 0 | 0.0 |
| AC#56 | 5 | 0.7 | - | - | - | - | - | - |
| AC#58 | 6 | 0.0 | - | - | - | - | - | - |
| AC#59 | 4 | 0.0 | 156 | 0 | 0.0 | - | - | - |
| AC#60 | 4 | 7.7 | 1404 | 133 | 9.5 | 126 | 21 | 16.7 |
| AC#61 | 4 | - | - | - | - | - | - | - |
| AC#62 | 5 | 0.0 | 84 | 1 | 1.2 | 1 | 1 | 100.0 |
| AC#63 | 7 | 0.0 | - | - | - | - | - | - |
| AC#64 | 6 | 0.0 | 1429 | 0 | 0.0 | - | - | - |
| AC#65 | 3 | 3.6 | 136 | 1 | 0.7 | 1 | 0 | 0.0 |
| AC#66 | 5 | 0.0 | 608 | 0 | 0.0 | - | - | - |
| AC#67 | 6 | 1.8 | 640 | 8 | 1.3 | 8 | 2 | 25.0 |
| AC#68 | 5 | 0.0 | 83 | 0 | 0.0 | - | - | - |
| AC#69 | 5 | 0.0 | - | - | - | - | - | - |
| AC#71 | 6 | 0.0 | 1465 | 1 | 0.1 | - | - | - |
| AC#72 | 6 | 0.0 | 332 | 0 | 0.0 | - | - | - |
| AC#73 | 5 | 4.9 | 248 | 0 | 0.0 | - | - | - |
| AC#75 | 4 | 3.0 | 167 | 0 | 0.0 | - | - | - |
| AC#76 | 6 | 0.0 | - | - | - | - | - | - |
| AC#78 | 4 | 0.0 | 246 | 0 | 0.0 | - | - | - |
| AC#79 | 1 | 0.0 | 132 | 1 | 0.8 | 1 | 0 | 0.0 |
| AC#81 | 5 | 0.0 | 162 | 0 | 0.0 | - | - | - |
| AC#82 | 6 | 0.0 | 245 | 0 | 0.0 | - | - | - |
| AC#83 | 5 | 0.0 | 336 | 0 | 0.0 | - | - | - |
| AC#84 | 6 | 0.8 | - | - | - | - | - | - |
| AC#85 | 5 | 0.0 | 270 | 0 | 0.0 | - | - | - |
| AC#86 | 7 | 0.0 | 872 | 2 | 0.2 | 2 | 2 | 100.0 |
| AC#87 | 7 | 0.0 | 355 | 0 | 0.0 | - | - | - |
| AC#88 | 6 | 0.2 | 419 | 16 | 3.8 | 16 | 7 | 43.8 |
| AC#89 | 5 | 0.2 | 306 | 2 | 0.7 | 2 | 0 | 0.0 |
| AC#90 | 6 | 0.6 | 1023 | 18 | 1.8 | 18 | 3 | 16.7 |
| AC#91 | 4 | 5.5 | 608 | 1 | 0.2 | 1 | 0 | 0.0 |
| AC#92 | 6 | 4.0 | - | - | - | - | - | - |
| AC#93 | 3 | - | 330 | 2 | 0.6 | 2 | 0 | 0.0 |
| AC#94 | 3 | 0.2 | 570 | 3 | 0.5 | 3 | 1 | 33.3 |
| AC#95 | 7 | 0.4 | 167 | 1 | 0.6 | 1 | 0 | 0.0 |
| AC#96 | 4 | 15.0 | 293 | 2 | 0.7 | 2 | 2 | 100.0 |
| AC#97 | 5 | - | 52 | 0 | 0.0 | - | - | - |
| AC#98 | 3 | 2.0 | 161 | 0 | 0.0 | - | - | - |
| AC#99 | 4 | 0.3 | 256 | 0 | 0.0 | - | - | - |
| AC#100 | 7 | 0.0 | 144 | 0 | 0.0 | - | - | - |
| AC#101 | 6 | 0.0 | 647 | 0 | 0.0 | - | - | - |
| AC#102 | 5 | 1.5 | - | - | - | - | - | - |
| AC#103 | 5 | 0.6 | 401 | 0 | 0.0 | - | - | - |
| AC#104 | 5 | 0.0 | 62 | 0 | 0.0 | - | - | - |
| AC#105 | 4 | 0.7 | 420 | 0 | 0.0 | - | - | - |
| AC#106 | 5 | 0.0 | 162 | 0 | 0.0 | - | - | - |
| AC#107 | 4 | 2.1 | 668 | 5 | 0.7 | 5 | 1 | 20.0 |
| AC#108 | 5 | 0.2 | 166 | 0 | 0.0 | - | - | - |
| AC#110 | 5 | 0.0 | 460 | 0 | 0.0 | - | - | - |
| AC#111 | 7 | 0.0 | 84 | 0 | 0.0 | - | - | - |
| AC#112 | 6 | 0.0 | - | - | - | - | - | - |
| AC#114 | 4 | 20.2 | 588 | 28 | 4.8 | 28 | 8 | 28.6 |
| AC#115 | 4 | 1.5 | - | - | - | - | - | - |
| AC#116 | 3 | 26.4 | - | - | - | - | - | - |
| AC#118 | 5 | 0.0 | - | - | - | - | - | - |
| AC#121 | 5 | 1.2 | - | - | - | - | - | - |
| AC#122 | 7 | 0.0 | 252 | 0 | 0.0 | - | - | - |
| AC#125 | 7 | 4.0 | 215 | 0 | 0.0 | - | - | - |
| AC#126 | 5 | 0.0 | - | - | - | - | - | - |
| AC#127 | 5 | - | - | - | - | - | - | - |
| AC#128 | 7 | 0.9 | - | - | - | - | - | - |
| AC#129 | 6 | 0.0 | - | - | - | - | - | - |
| AC#131 | 3 | 23.2 | - | - | - | - | - | - |
| AC#132 | 5 | 1.1 | 252 | 1 | 0.4 | - | - | - |
| AC#134 | 7 | 0.0 | 420 | 0 | 0.0 | - | - | - |
| AC#136 | 6 | - | - | - | - | - | - | - |
| AC#137 | 7 | 0.0 | - | - | - | - | - | - |
| AC#138 | 6 | 0.9 | 252 | 1 | 0.4 | 1 | 0 | 0.0 |
| AC#139 | 4 | 2.3 | - | - | - | - | - | - |
| AC#140 | 7 | 0.0 | - | - | - | - | - | - |
| AC#141 | 4 | 0.0 | - | - | - | - | - | - |
| AC#142 | 7 | 0.0 | - | - | - | - | - | - |
| AC#143 | 5 | 0.9 | - | - | - | - | - | - |
| AC#144 | 5 | 0.0 | 210 | 3 | 1.4 | 3 | 1 | 33.3 |
| AC#145 | 4 | 0.0 | 588 | 4 | 0.7 | 4 | 1 | 25.0 |
| AC#146 | 5 | - | 144 | 0 | 0.0 | - | - | - |
| AC#147 | 5 | 0.0 | - | - | - | - | - | - |
| AC#148 | 6 | 0.0 | - | - | - | - | - | - |
| AC#149 | 7 | 1.1 | - | - | - | - | - | - |
| AC#150 | 7 | - | - | - | - | - | - | - |
| AC#151 | 5 | 0.0 | 1645 | 1 | 0.1 | 1 | 1 | 100.0 |
| AC#152 | 5 | 0.0 | 698 | 4 | 0.6 | 4 | 1 | 25.0 |
| AC#154 | 6 | 9.1 | - | - | - | - | - | - |
| AC#155 | 5 | - | - | - | - | - | - | - |
| * The data of the number of the heterozygous HS loci were sourced from Kuniyoshi et al., 2024. | | | | | | | | |

| Table S2 (1/2). Genotype of the plants in the AC_1_ population. | | | | | | | | | | | |
| --- | --- | --- | --- | --- | --- | --- | --- | --- | --- | --- | --- |
| Variety | DNA marker | | |  |  |  |  |  |  |  |  |
|  | c1-7.42 | UH1-d2 | UH2-u4 | UH3-d1 | UH4-d1 | c5-1.25 | UH5-u1 | UH6-u2 | UH7-u2 | c8-1.7 | UH8-d1 |
| PL9 | *S* | *S* | *S* | *S* | *S* | *S* | *S* | *S* | *S* | *S* | *S* |
| WK21 | *G* | *G* | *G* | *G* | *G* | *G* | *G* | *G* | *G* | *G* | *G* |
| WK21-2X/PL9-2X F_1_ | *H* | *H* | *H* | *H* | *H* | *H* | *H* | *H* | *H* | *H* | *H* |
| AC#1 | *G* | *G* | *H* | *H* | *H* | *H* | *H* | *S* | *H* | *H* | *H* |
| AC#2 | *S* | *S* | *H* | *H* | *H* | *H* | *H* | *H* | *H* | *H* | *H* |
| AC#3 | *G* | *S* | *H* | *G* | *S* | *S* | *H* | *H* | *H* | *H* | *H* |
| AC#4 | *G* | *H* | *H* | *G* | *S* | *S* | *H* | *H* | *G* | *H* | *G* |
| AC#5 | *G* | *H* | *H* | *H* | *G* | *H* | *H* | *H* | *H* | *G* | *H* |
| AC#6 | *H* | *H* | *S* | *H* | *H* | *G* | *H* | *S* | *H* | *H* | *H* |
| AC#7 | *H* | *H* | *H* | *G* | *S* | *H* | *H* | *S* | *H* | *H* | *H* |
| AC#8 | *H* | *H* | *S* | *H* | *G* | *H* | *H* | *G* | *H* | *H* | *H* |
| AC#9 | *S* | *S* | *H* | *H* | *H* | *H* | *H* | *H* | *H* | *G* | *H* |
| AC#11 | *H* | *H* | *H* | *H* | *H* | *S* | *S* | *S* | *H* | *H* | *H* |
| AC#12 | *S* | *H* | *G* | *H* | *H* | *H* | *S* | *G* | *H* | *H* | *S* |
| AC#13 | *H* | *H* | *H* | *H* | *H* | *H* | *H* | *H* | *H* | *H* | *G* |
| AC#14 | *S* | *S* | *H* | *S* | *H* | *S* | *H* | *H* | *H* | *H* | *H* |
| AC#15 | *S* | *S* | *H* | *G* | *H* | *S* | *S* | *H* | *H* | *H* | *H* |
| AC#16 | *S* | *S* | *H* | *H* | *S* | *H* | *H* | *H* | *H* | *H* | *G* |
| AC#17 | *G* | *G* | *H* | *H* | *S* | *S* | *S* | *S* | *H* | *H* | *H* |
| AC#18 | *S* | *H* | *H* | *H* | *H* | *H* | *H* | *H* | *H* | *H* | *S* |
| AC#19 | *G* | *H* | *H* | *H* | *G* | *H* | *H* | *H* | *H* | *G* | *H* |
| AC#20 | *G* | *H* | *H* | *H* | *S* | *S* | *S* | *H* | *H* | *H* | *H* |
| AC#21 | *S* | *G* | *H* | *H* | *S* | *H* | *H* | *S* | *H* | *G* | *H* |
| AC#22 | *H* | *H* | *H* | *H* | *S* | *H* | *H* | *S* | *H* | *H* | *H* |
| AC#23 | *H* | *S* | *S* | *H* | *H* | *H* | *H* | *H* | *H* | *G* | *H* |
| AC#24 | *S* | *S* | *H* | *H* | *H* | *G* | *H* | *G* | *H* | *G* | *S* |
| AC#25 | *H* | *G* | *H* | *H* | *H* | *H* | *S* | *S* | *H* | *H* | *H* |
| AC#26 | *H* | *G* | *H* | *G* | *S* | *H* | *H* | *H* | *S* | *H* | *S* |
| AC#27 | *S* | *H* | *H* | *H* | *H* | *S* | *S* | *H* | *S* | *H* | *S* |
| AC#28 | *S* | *S* | *H* | *H* | *S* | *H* | *S* | *H* | *H* | *G* | *H* |
| AC#30 | *H* | *H* | *H* | *S* | *G* | *H* | *H* | *H* | *H* | *G* | *H* |
| AC#32 | *H* | *H* | *H* | *G* | *S* | *S* | *S* | *S* | *H* | *H* | *H* |
| AC#33 | *S* | *S* | *H* | *H* | *H* | *S* | *S* | *G* | *H* | *G* | *S* |
| AC#34 | *G* | *S* | *H* | *H* | *H* | *H* | *H* | *S* | *G* | *H* | *H* |
| AC#35 | *H* | *H* | *S* | *H* | *H* | *H* | *G* | *G* | *H* | *S* | *H* |
| AC#37 | *G* | *H* | *H* | *H* | *S* | *S* | *H* | *S* | *S* | *H* | *H* |
| AC#38 | *H* | *H* | *H* | *G* | *H* | *H* | *H* | *H* | *H* | *S* | *H* |
| AC#39 | *S* | *S* | *H* | *H* | *S* | *H* | *S* | *G* | *H* | *H* | *G* |
| AC#40 | *S* | *S* | *H* | *H* | *H* | *H* | *H* | *G* | *H* | *H* | *G* |
| AC#41 | *H* | *H* | *H* | *H* | *H* | *H* | *S* | *G* | *H* | *H* | *H* |
| AC#42 | *H* | *H* | *H* | *H* | *H* | *S* | *H* | *H* | *H* | *S* | *S* |
| AC#43 | *H* | *H* | *H* | *H* | *S* | *H* | *H* | *H* | *H* | *H* | *G* |
| AC#44 | *H* | *S* | *H* | *S* | *S* | *H* | *H* | *H* | *S* | *S* | *H* |
| AC#45 | *H* | *H* | *H* | *H* | *H* | *G* | *H* | *G* | *H* | *H* | *G* |
| AC#46 | *H* | *G* | *H* | *S* | *S* | *S* | *H* | *H* | *H* | *H* | *H* |
| AC#47 | *S* | *S* | *S* | *S* | *H* | *H* | *H* | *H* | *S* | *H* | *S* |
| AC#48 | *H* | *H* | *H* | *H* | *H* | *H* | *H* | *H* | *H* | *H* | *H* |
| AC#49 | *H* | *H* | *H* | *G* | *H* | *H* | *H* | *H* | *H* | *G* | *H* |
| AC#50 | *H* | *H* | *H* | *H* | *S* | *S* | *H* | *S* | *H* | *H* | *G* |
| AC#51 | *S* | *S* | *H* | *S* | *H* | *H* | *H* | *H* | *H* | *H* | *H* |
| AC#52 | *H* | *H* | *S* | *H* | *H* | *S* | *H* | *H* | *H* | *G* | *G* |
| AC#53 | *G* | *H* | *S* | *H* | *S* | *S* | *H* | *S* | *H* | *H* | *H* |
| AC#54 | *H* | *G* | *H* | *H* | *S* | *H* | *H* | *S* | *H* | *H* | *H* |
| AC#56 | *H* | *S* | *S* | *S* | *H* | *H* | *H* | *H* | *H* | *H* | *H* |
| AC#58 | *G* | *H* | *S* | *H* | *S* | *S* | *S* | *H* | *H* | *H* | *H* |
| AC#59 | *S* | *S* | *S* | *H* | *H* | *S* | *S* | *H* | *G* | *H* | *G* |
| AC#60 | *H* | *S* | *H* | *G* | *H* | *S* | *S* | *S* | *H* | *H* | *S* |
| AC#61 | *S* | *G* | *H* | *H* | *G* | *H* | *-* | *S* | *H* | *H* | *S* |
| AC#62 | *H* | *H* | *H* | *G* | *H* | *H* | *H* | *H* | *S* | *G* | *H* |
| AC#63 | *S* | *G* | *S* | *S* | *H* | *S* | *H* | *S* | *H* | *S* | *H* |
| AC#64 | *H* | *G* | *H* | *H* | *S* | *H* | *H* | *S* | *H* | *H* | *H* |
| AC#65 | *H* | *G* | *H* | *S* | *S* | *S* | *H* | *H* | *H* | *H* | *H* |
| AC#66 | *H* | *H* | *H* | *H* | *S* | *H* | *H* | *H* | *H* | *G* | *H* |
| AC#67 | *H* | *H* | *H* | *H* | *S* | *H* | *H* | *S* | *H* | *H* | *H* |
| AC#68 | *H* | *H* | *H* | *G* | *H* | *S* | *H* | *H* | *G* | *S* | *H* |
| AC#69 | *G* | *G* | *H* | *G* | *S* | *S* | *S* | *H* | *H* | *H* | *H* |
| AC#71 | *H* | *H* | *H* | *H* | *S* | *H* | *H* | *H* | *H* | *H* | *G* |
| AC#72 | *G* | *H* | *S* | *H* | *S* | *S* | *S* | *H* | *H* | *H* | *H* |
| AC#73 | *H* | *S* | *H* | *H* | *S* | *S* | *H* | *G* | *H* | *G* | *G* |
| AC#75 | *H* | *H* | *S* | *H* | *S* | *H* | *H* | *H* | *H* | *H* | *H* |
| AC#76 | *G* | *G* | *H* | *H* | *S* | *S* | *S* | *S* | *H* | *H* | *H* |
| AC#78 | *S* | *S* | *H* | *S* | *H* | *H* | *H* | *H* | *H* | *H* | *H* |
| AC#79 | *H* | *H* | *H* | *H* | *S* | *0* | *H* | *S* | *H* | *H* | *S* |
| AC#81 | *H* | *H* | *S* | *S* | *S* | *H* | *H* | *H* | *H* | *H* | *H* |
| AC#82 | *H* | *H* | *H* | *H* | *S* | *H* | *H* | *G* | *S* | *G* | *H* |
| AC#83 | *H* | *S* | *S* | *H* | *S* | *H* | *H* | *H* | *H* | *H* | *H* |
| AC#84 | *H* | *H* | *H* | *H* | *S* | *H* | *H* | *G* | *S* | *G* | *H* |
| AC#85 | *H* | *H* | *S* | *H* | *H* | *H* | *H* | *S* | *G* | *H* | *H* |
| AC#86 | *H* | *G* | *H* | *G* | *H* | *G* | *H* | *H* | *H* | *H* | *H* |
| AC#87 | *G* | *H* | *H* | *H* | *H* | *S* | *S* | *H* | *H* | *S* | *H* |
| AC#88 | *G* | *G* | *H* | *H* | *H* | *H* | *S* | *G* | *H* | *H* | *H* |
| AC#89 | *H* | *H* | *H* | *H* | *S* | *H* | *H* | *H* | *H* | *G* | *H* |
| AC#90 | *G* | *S* | *H* | *G* | *S* | *S* | *S* | *H* | *H* | *H* | *H* |
| AC#91 | *H* | *H* | *H* | *H* | *H* | *H* | *H* | *S* | *H* | *H* | *S* |
| AC#92 | *S* | *H* | *G* | *H* | *H* | *S* | *H* | *H* | *H* | *H* | *G* |
| AC#93 | *H* | *H* | *S* | *H* | *S* | *H* | *H* | *H* | *H* | *H* | *H* |
| AC#94 | *G* | *H* | *S* | *H* | *S* | *S* | *H* | *S* | *H* | *H* | *H* |
| AC#95 | *H* | *H* | *H* | *S* | *H* | *H* | *H* | *G* | *H* | *G* | *S* |
| AC#96 | *G* | *G* | *H* | *G* | *H* | *H* | *H* | *H* | *H* | *H* | *H* |
| AC#97 | *H* | *S* | *S* | *S* | *H* | *H* | *H* | *H* | *H* | *H* | *H* |
| AC#98 | *H* | *G* | *H* | *H* | *S* | *H* | *H* | *S* | *G* | *G* | *H* |
| AC#99 | *S* | *S* | *S* | *H* | *S* | *S* | *S* | *H* | *H* | *H* | *S* |
| AC#100 | *H* | *H* | *H* | *G* | *H* | *S* | *S* | *H* | *H* | *H* | *H* |
| AC#101 | *G* | *H* | *H* | *G* | *H* | *H* | *H* | *H* | *G* | *G* | *H* |
| AC#102 | *H* | *H* | *S* | *H* | *S* | *S* | *S* | *H* | *H* | *H* | *S* |
| AC#103 | *S* | *S* | *H* | *H* | *S* | *H* | *H* | *H* | *H* | *H* | *S* |
| AC#104 | *H* | *S* | *H* | *H* | *H* | *G* | *G* | *H* | *H* | *H* | *G* |
| AC#105 | *S* | *G* | *H* | *H* | *S* | *H* | *H* | *S* | *G* | *H* | *H* |
| AC#106 | *H* | *H* | *S* | *H* | *S* | *S* | *S* | *H* | *H* | *H* | *S* |
| AC#107 | *S* | *S* | *S* | *H* | *H* | *S* | *H* | *S* | *G* | *H* | *S* |
| AC#108 | *H* | *H* | *G* | *H* | *H* | *H* | *H* | *G* | *H* | *H* | *H* |
| AC#110 | *H* | *G* | *H* | *H* | *H* | *H* | *H* | *S* | *H* | *H* | *H* |
| AC#111 | *G* | *H* | *H* | *H* | *S* | *H* | *H* | *H* | *H* | *H* | *H* |
| AC#112 | *H* | *S* | *H* | *S* | *H* | *G* | *H* | *H* | *H* | *G* | *H* |
| AC#114 | *H* | *S* | *H* | *H* | *S* | *S* | *H* | *H* | *H* | *G* | *H* |
| AC#115 | *G* | *-* | *S* | *S* | *H* | *G* | *G* | *H* | *H* | *H* | *H* |
| AC#116 | *H* | *H* | *H* | *H* | *S* | *H* | *H* | *G* | *S* | *H* | *H* |
| AC#118 | *H* | *H* | *G* | *H* | *H* | *H* | *H* | *H* | *H* | *H* | *H* |
| AC#121 | *H* | *G* | *H* | *H* | *S* | *H* | *H* | *S* | *H* | *H* | *H* |
| AC#122 | *S* | *S* | *G* | *H* | *S* | *H* | *H* | *H* | *H* | *H* | *H* |
| AC#125 | *H* | *H* | *H* | *H* | *H* | *H* | *H* | *H* | *H* | *G* | *G* |
| AC#126 | *S* | *S* | *H* | *H* | *H* | *S* | *H* | *H* | *H* | *S* | *S* |
| AC#127 | *G* | *H* | *H* | *H* | *H* | *S* | *S* | *H* | *S* | *G* | *G* |
| AC#128 | *S* | *S* | *H* | *H* | *S* | *G* | *H* | *H* | *H* | *H* | *G* |
| AC#129 | *G* | *S* | *S* | *H* | *H* | *S* | *S* | *S* | *H* | *H* | *S* |
| AC#131 | *H* | *H* | *S* | *H* | *H* | *H* | *G* | *G* | *H* | *G* | *H* |
| AC#132 | *S* | *S* | *S* | *H* | *S* | *H* | *H* | *H* | *H* | *H* | *S* |
| AC#134 | *S* | *S* | *G* | *H* | *S* | *H* | *H* | *-* | *H* | *H* | *H* |
| AC#136 | *S* | *S* | *S* | *H* | *H* | *G* | *H* | *H* | *H* | *G* | *H* |
| AC#137 | *S* | *S* | *H* | *H* | *H* | *H* | *H* | *H* | *H* | *H* | *H* |
| AC#138 | *H* | *H* | *S* | *H* | *H* | *H* | *H* | *H* | *H* | *H* | *S* |
| AC#139 | *S* | *S* | *H* | *S* | *H* | *S* | *H* | *H* | *G* | *H* | *H* |
| AC#140 | *H* | *H* | *H* | *S* | *G* | *G* | *H* | *H* | *H* | *S* | *H* |
| AC#141 | *G* | *H* | *H* | *H* | *S* | *S* | *H* | *S* | *S* | *H* | *H* |
| AC#142 | *H* | *H* | *H* | *H* | *H* | *H* | *H* | *H* | *H* | *H* | *H* |
| AC#143 | *S* | *S* | *H* | *S* | *H* | *H* | *H* | *G* | *H* | *H* | *G* |
| AC#144 | *S* | *S* | *H* | *S* | *H* | *H* | *H* | *G* | *H* | *H* | *G* |
| AC#145 | *S* | *S* | *H* | *H* | *H* | *H* | *H* | *H* | *-* | *H* | *H* |
| AC#146 | *S* | *H* | *S* | *H* | *S* | *S* | *H* | *H* | *H* | *H* | *S* |
| AC#147 | *G* | *H* | *H* | *G* | *S* | *S* | *H* | *H* | *H* | *S* | *H* |
| AC#148 | *H* | *H* | *H* | *S* | *S* | *S* | *S* | *H* | *H* | *H* | *H* |
| AC#149 | *G* | *H* | *H* | *H* | *H* | *S* | *H* | *H* | *H* | *H* | *H* |
| AC#150 | *H* | *H* | *H* | *H* | *H* | *S* | *H* | *S* | *H* | *H* | *G* |
| AC#151 | *H* | *H* | *S* | *H* | *H* | *H* | *H* | *H* | *H* | *H* | *H* |
| AC#152 | *S* | *H* | *G* | *H* | *H* | *H* | *S* | *G* | *G* | *H* | *S* |
| AC#154 | *H* | *H* | *H* | *H* | *H* | *H* | *H* | *S* | *H* | *G* | *S* |
| AC#155 | *H* | *H* | *H* | *G* | *H* | *H* | *H* | *G* | *H* | *S* | *G* |

| Table S2 (2/2). Genotype of the plants in the AC_1_ population. | | | | | | | | | | |
| --- | --- | --- | --- | --- | --- | --- | --- | --- | --- | --- |
| Plant  materials |  |  |  |  |  |  |  |  |  |  |
|  | UH9-d1 | c9-19.9 | UH10-d3 | c10-22.2 | c11-5.0 | UH11-d1 | c11-25.0 | C12-2.0 | UH12-d1 | c12-25.1 |
| PL9 | *S* | *S* | *S* | *S* | *S* | *S* | *S* | *S* | *S* | *S* |
| WK21 | *G* | *G* | *G* | *G* | *G* | *G* | *G* | *G* | *G* | *G* |
| WK21-2X/PL9-2X F_1_ | *H* | *H* | *H* | *H* | *H* | *H* | *H* | *H* | *H* | *H* |
| AC#1 | *H* | *H* | *H* | *H* | *H* | *H* | *H* | *S* | *H* | *H* |
| AC#2 | *H* | *H* | *H* | *H* | *H* | *H* | *H* | *H* | *H* | *H* |
| AC#3 | *H* | *H* | *H* | *H* | *H* | *H* | *H* | *G* | *G* | *H* |
| AC#4 | *H* | *H* | *H* | *H* | *H* | *H* | *H* | *H* | *G* | *H* |
| AC#5 | *-* | *G* | *H* | *H* | *H* | *H* | *H* | *H* | *H* | *H* |
| AC#6 | *H* | *H* | *S* | *H* | *H* | *H* | *H* | *H* | *H* | *H* |
| AC#7 | *H* | *H* | *H* | *H* | *G* | *G* | *H* | *H* | *H* | *H* |
| AC#8 | *G* | *H* | *H* | *H* | *H* | *H* | *H* | *H* | *H* | *S* |
| AC#9 | *G* | *H* | *H* | *H* | *H* | *H* | *H* | *H* | *H* | *S* |
| AC#11 | *G* | *G* | *H* | *G* | *H* | *H* | *H* | *H* | *G* | *G* |
| AC#12 | *H* | *-* | *H* | *G* | *H* | *H* | *H* | *G* | *H* | *H* |
| AC#13 | *H* | *H* | *H* | *G* | *H* | *H* | *H* | *H* | *G* | *G* |
| AC#14 | *H* | *H* | *H* | *H* | *H* | *H* | *S* | *H* | *H* | *H* |
| AC#15 | *H* | *H* | *H* | *S* | *H* | *H* | *G* | *G* | *H* | *H* |
| AC#16 | *G* | *H* | *H* | *H* | *H* | *H* | *H* | *G* | *H* | *H* |
| AC#17 | *-* | *H* | *H* | *S* | *H* | *H* | *S* | *H* | *H* | *-* |
| AC#18 | *H* | *H* | *H* | *H* | *H* | *H* | *S* | *H* | *H* | *H* |
| AC#19 | *G* | *G* | *H* | *H* | *H* | *H* | *H* | *H* | *H* | *S* |
| AC#20 | *G* | *H* | *H* | *H* | *H* | *H* | *H* | *H* | *H* | *H* |
| AC#21 | *G* | *G* | *H* | *H* | *H* | *H* | *H* | *H* | *H* | *H* |
| AC#22 | *G* | *H* | *H* | *H* | *H* | *G* | *G* | *H* | *H* | *H* |
| AC#23 | *H* | *G* | *H* | *H* | *H* | *H* | *H* | *H* | *G* | *G* |
| AC#24 | *H* | *G* | *H* | *G* | *G* | *G* | *H* | *G* | *H* | *H* |
| AC#25 | *H* | *H* | *S* | *G* | *S* | *H* | *G* | *H* | *H* | *H* |
| AC#26 | *H* | *H* | *H* | *H* | *G* | *H* | *S* | *H* | *S* | *S* |
| AC#27 | *H* | *H* | *G* | *S* | *H* | *H* | *H* | *H* | *H* | *H* |
| AC#28 | *H* | *G* | *H* | *H* | *H* | *H* | *S* | *H* | *H* | *S* |
| AC#30 | *G* | *G* | *H* | *S* | *G* | *G* | *H* | *S* | *H* | *H* |
| AC#32 | *H* | *H* | *H* | *H* | *G* | *H* | *H* | *H* | *H* | *H* |
| AC#33 | *H* | *G* | *H* | *H* | *H* | *H* | *H* | *G* | *H* | *H* |
| AC#34 | *H* | *H* | *H* | *G* | *H* | *H* | *H* | *S* | *S* | *H* |
| AC#35 | *H* | *S* | *S* | *G* | *G* | *G* | *G* | *H* | *H* | *H* |
| AC#37 | *H* | *H* | *S* | *H* | *H* | *H* | *H* | *H* | *G* | *G* |
| AC#38 | *G* | *S* | *S* | *G* | *H* | *H* | *H* | *H* | *H* | *G* |
| AC#39 | *G* | *H* | *H* | *H* | *H* | *H* | *S* | *H* | *H* | *H* |
| AC#40 | *H* | *H* | *H* | *H* | *H* | *H* | *S* | *S* | *S* | *S* |
| AC#41 | *H* | *H* | *H* | *H* | *H* | *H* | *H* | *H* | *H* | *H* |
| AC#42 | *S* | *S* | *H* | *H* | *H* | *H* | *H* | *H* | *H* | *H* |
| AC#43 | *-* | *H* | *S* | *S* | *H* | *H* | *H* | *G* | *H* | *S* |
| AC#44 | *H* | *S* | *H* | *H* | *S* | *S* | *G* | *H* | *H* | *H* |
| AC#45 | *H* | *H* | *H* | *H* | *G* | *H* | *H* | *H* | *H* | *G* |
| AC#46 | *H* | *H* | *H* | *H* | *H* | *H* | *H* | *H* | *H* | *H* |
| AC#47 | *G* | *H* | *H* | *H* | *H* | *H* | *S* | *S* | *S* | *H* |
| AC#48 | *S* | *H* | *H* | *G* | *H* | *H* | *H* | *H* | *H* | *G* |
| AC#49 | *G* | *G* | *G* | *H* | *G* | *G* | *H* | *H* | *H* | *S* |
| AC#50 | *H* | *H* | *S* | *H* | *H* | *H* | *H* | *H* | *H* | *H* |
| AC#51 | *H* | *H* | *H* | *S* | *H* | *H* | *H* | *H* | *H* | *H* |
| AC#52 | *G* | *G* | *H* | *H* | *H* | *H* | *S* | *H* | *H* | *S* |
| AC#53 | *H* | *H* | *H* | *G* | *H* | *G* | *G* | *H* | *H* | *-* |
| AC#54 | *G* | *H* | *S* | *H* | *H* | *H* | *G* | *H* | *H* | *H* |
| AC#56 | *H* | *H* | *H* | *H* | *G* | *G* | *H* | *H* | *H* | *H* |
| AC#58 | *G* | *H* | *S* | *H* | *G* | *H* | *H* | *G* | *G* | *H* |
| AC#59 | *G* | *H* | *H* | *H* | *H* | *H* | *H* | *H* | *H* | *H* |
| AC#60 | *H* | *H* | *H* | *H* | *H* | *H* | *H* | *H* | *H* | *H* |
| AC#61 | *H* | *H* | *S* | *H* | *G* | *G* | *S* | *H* | *H* | *H* |
| AC#62 | *H* | *G* | *G* | *H* | *H* | *H* | *H* | *H* | *H* | *H* |
| AC#63 | *G* | *S* | *H* | *G* | *H* | *H* | *G* | *G* | *G* | *G* |
| AC#64 | *G* | *H* | *S* | *H* | *H* | *H* | *G* | *H* | *H* | *H* |
| AC#65 | *H* | *H* | *H* | *H* | *H* | *H* | *H* | *H* | *H* | *H* |
| AC#66 | *H* | *G* | *H* | *H* | *H* | *-* | *H* | *S* | *H* | *H* |
| AC#67 | *G* | *H* | *H* | *H* | *H* | *H* | *G* | *H* | *H* | *H* |
| AC#68 | *-* | *S* | *H* | *H* | *H* | *H* | *H* | *H* | *H* | *H* |
| AC#69 | *H* | *H* | *H* | *G* | *H* | *H* | *H* | *H* | *G* | *H* |
| AC#71 | *H* | *H* | *S* | *S* | *H* | *H* | *H* | *G* | *H* | *S* |
| AC#72 | *G* | *H* | *S* | *H* | *H* | *H* | *H* | *G* | *G* | *H* |
| AC#73 | *G* | *G* | *H* | *H* | *H* | *H* | *H* | *H* | *S* | *S* |
| AC#75 | *H* | *H* | *H* | *H* | *H* | *H* | *H* | *S* | *H* | *H* |
| AC#76 | *H* | *H* | *H* | *S* | *H* | *H* | *S* | *H* | *H* | *H* |
| AC#78 | *H* | *H* | *H* | *H* | *H* | *H* | *H* | *S* | *S* | *H* |
| AC#79 | *G* | *H* | *H* | *H* | *H* | *G* | *H* | *H* | *H* | *G* |
| AC#81 | *H* | *H* | *H* | *H* | *H* | *H* | *G* | *H* | *H* | *G* |
| AC#82 | *G* | *G* | *S* | *H* | *H* | *G* | *H* | *H* | *H* | *H* |
| AC#83 | *H* | *H* | *H* | *H* | *H* | *H* | *S* | *H* | *H* | *H* |
| AC#84 | *G* | *G* | *S* | *H* | *H* | *H* | *H* | *H* | *H* | *H* |
| AC#85 | *H* | *H* | *S* | *G* | *G* | *G* | *G* | *H* | *G* | *G* |
| AC#86 | *H* | *H* | *H* | *G* | *H* | *H* | *H* | *H* | *H* | *H* |
| AC#87 | *H* | *S* | *H* | *G* | *S* | *H* | *S* | *H* | *H* | *H* |
| AC#88 | *H* | *H* | *G* | *G* | *H* | *H* | *H* | *H* | *H* | *H* |
| AC#89 | *H* | *G* | *H* | *H* | *H* | *H* | *H* | *S* | *H* | *H* |
| AC#90 | *H* | *H* | *H* | *H* | *H* | *H* | *H* | *G* | *G* | *H* |
| AC#91 | *H* | *H* | *H* | *H* | *H* | *H* | *G* | *H* | *H* | *H* |
| AC#92 | *H* | *H* | *H* | *S* | *H* | *H* | *H* | *H* | *G* | *H* |
| AC#93 | *H* | *H* | *H* | *H* | *H* | *G* | *H* | *S* | *H* | *H* |
| AC#94 | *H* | *H* | *H* | *G* | *H* | *G* | *G* | *H* | *H* | *H* |
| AC#95 | *H* | *H* | *H* | *G* | *G* | *G* | *H* | *H* | *H* | *H* |
| AC#96 | *H* | *G* | *H* | *H* | *H* | *H* | *H* | *H* | *H* | *H* |
| AC#97 | *H* | *H* | *H* | *H* | *H* | *H* | *H* | *H* | *H* | *H* |
| AC#98 | *H* | *H* | *H* | *G* | *H* | *G* | *G* | *S* | *H* | *H* |
| AC#99 | *H* | *H* | *H* | *H* | *H* | *H* | *G* | *H* | *H* | *H* |
| AC#100 | *H* | *H* | *S* | *H* | *H* | *H* | *H* | *H* | *H* | *H* |
| AC#101 | *H* | *H* | *H* | *G* | *H* | *H* | *H* | *G* | *G* | *H* |
| AC#102 | *H* | *G* | *H* | *H* | *H* | *H* | *H* | *H* | *H* | *H* |
| AC#103 | *H* | *H* | *H* | *H* | *H* | *H* | *S* | *S* | *H* | *H* |
| AC#104 | *H* | *H* | *H* | *H* | *H* | *H* | *H* | *H* | *H* | *H* |
| AC#105 | *H* | *H* | *H* | *H* | *G* | *G* | *H* | *G* | *H* | *H* |
| AC#106 | *H* | *G* | *H* | *H* | *H* | *H* | *H* | *H* | *H* | *H* |
| AC#107 | *G* | *G* | *H* | *H* | *G* | *H* | *H* | *H* | *H* | *H* |
| AC#108 | *H* | *H* | *H* | *H* | *H* | *H* | *H* | *H* | *H* | *H* |
| AC#110 | *H* | *H* | *S* | *H* | *H* | *H* | *H* | *H* | *H* | *H* |
| AC#111 | *H* | *H* | *H* | *H* | *H* | *H* | *H* | *G* | *H* | *H* |
| AC#112 | *H* | *H* | *H* | *G* | *G* | *G* | *H* | *H* | *G* | *H* |
| AC#114 | *H* | *H* | *G* | *G* | *H* | *H* | *H* | *S* | *H* | *G* |
| AC#115 | *H* | *H* | *H* | *H* | *H* | *H* | *H* | *H* | *H* | *H* |
| AC#116 | *G* | *H* | *S* | *H* | *H* | *G* | *S* | *G* | *G* | *S* |
| AC#118 | *H* | *H* | *H* | *H* | *H* | *H* | *H* | *H* | *H* | *H* |
| AC#121 | *G* | *H* | *S* | *H* | *H* | *G* | *H* | *H* | *H* | *-* |
| AC#122 | *H* | *H* | *H* | *H* | *H* | *H* | *H* | *H* | *H* | *H* |
| AC#125 | *H* | *H* | *H* | *G* | *H* | *H* | *H* | *H* | *H* | *H* |
| AC#126 | *H* | *H* | *H* | *S* | *S* | *H* | *H* | *H* | *H* | *H* |
| AC#127 | *H* | *G* | *H* | *G* | *H* | *H* | *H* | *H* | *H* | *H* |
| AC#128 | *H* | *H* | *H* | *H* | *G* | *G* | *S* | *H* | *H* | *H* |
| AC#129 | *H* | *H* | *H* | *H* | *H* | *H* | *H* | *H* | *H* | *H* |
| AC#131 | *H* | *S* | *S* | *G* | *G* | *G* | *G* | *H* | *H* | *H* |
| AC#132 | *H* | *H* | *H* | *H* | *H* | *H* | *H* | *H* | *H* | *H* |
| AC#134 | *H* | *H* | *H* | *H* | *H* | *H* | *H* | *H* | *H* | *H* |
| AC#136 | *H* | *H* | *G* | *G* | *H* | *H* | *G* | *H* | *H* | *H* |
| AC#137 | *G* | *G* | *H* | *H* | *H* | *H* | *H* | *H* | *H* | *S* |
| AC#138 | *H* | *H* | *S* | *H* | *H* | *H* | *H* | *G* | *G* | *G* |
| AC#139 | *G* | *G* | *H* | *H* | *H* | *H* | *S* | *S* | *S* | *S* |
| AC#140 | *S* | *S* | *H* | *S* | *G* | *H* | *H* | *H* | *H* | *H* |
| AC#141 | *H* | *H* | *S* | *H* | *H* | *H* | *H* | *H* | *G* | *G* |
| AC#142 | *H* | *H* | *H* | *H* | *H* | *H* | *H* | *H* | *H* | *H* |
| AC#143 | *H* | *G* | *H* | *H* | *S* | *S* | *H* | *H* | *H* | *H* |
| AC#144 | *H* | *G* | *H* | *H* | *S* | *S* | *H* | *H* | *H* | *H* |
| AC#145 | *G* | *G* | *H* | *H* | *H* | *H* | *H* | *S* | *S* | *S* |
| AC#146 | *H* | *G* | *G* | *H* | *H* | *H* | *H* | *G* | *G* | *H* |
| AC#147 | *H* | *H* | *H* | *S* | *H* | *H* | *H* | *G* | *G* | *G* |
| AC#148 | *H* | *S* | *H* | *H* | *H* | *H* | *H* | *H* | *H* | *H* |
| AC#149 | *H* | *S* | *S* | *H* | *H* | *H* | *H* | *G* | *G* | *H* |
| AC#150 | *H* | *H* | *G* | *H* | *H* | *H* | *H* | *H* | *H* | *S* |
| AC#151 | *G* | *G* | *H* | *H* | *H* | *H* | *H* | *H* | *H* | *H* |
| AC#152 | *H* | *H* | *H* | *S* | *G* | *H* | *H* | *H* | *H* | *H* |
| AC#154 | *G* | *H* | *H* | *G* | *H* | *G* | *G* | *H* | *H* | *H* |
| AC#155 | *G* | *G* | *H* | *S* | *H* | *H* | *G* | *H* | *H* | *H* |

| Table S3. Ploidy analysis of the anther culture population derived from anther culture of the AC_1_ plants. | | | | | |
| --- | --- | --- | --- | --- | --- |
| No. | Plant ID | Relative ploidy  to PL9-2X | Relative ploidy  to WK21-2X | Relative ploidy  (Average) | Estimated ploidy |
| 1 | 5 | 0.59 | 0.51 | 0.55 | Haploid |
| 2 | 6 | 0.60 | 0.51 | 0.55 | Haploid |
| 3 | 9 | 0.59 | 0.51 | 0.54 | Haploid |
| 4 | 10 | 0.60 | 0.52 | 0.55 | Haploid |
| 5 | 16 | 0.57 | 0.49 | 0.53 | Haploid |
| 6 | 34 | 0.57 | 0.48 | 0.52 | Haploid |
| 7 | 51 | 0.68 | 0.57 | 0.62 | Haploid |
| 8 | 52 | 0.57 | 0.47 | 0.52 | Haploid |
| 9 | 59 | 0.55 | 0.46 | 0.50 | Haploid |
| 10 | 2 | 1.15 | 0.99 | 1.07 | Diploid |
| 11 | 3 | 1.15 | 0.99 | 1.07 | Diploid |
| 12 | 7 | 1.26 | 1.09 | 1.17 | Diploid |
| 13 | 11 | 1.17 | 1.01 | 1.08 | Diploid |
| 14 | 12 | 1.16 | 1.00 | 1.07 | Diploid |
| 15 | 13 | 1.17 | 1.00 | 1.08 | Diploid |
| 16 | 14 | 1.17 | 1.01 | 1.09 | Diploid |
| 17 | 15 | 1.22 | 1.05 | 1.13 | Diploid |
| 18 | 18 | 1.13 | 0.97 | 1.05 | Diploid |
| 19 | 19 | 1.15 | 0.99 | 1.06 | Diploid |
| 20 | 23 | 1.10 | 0.95 | 1.02 | Diploid |
| 21 | 24 | 1.14 | 0.98 | 1.06 | Diploid |
| 22 | 25 | 1.16 | 1.00 | 1.07 | Diploid |
| 23 | 32 | 1.09 | 0.90 | 0.99 | Diploid |
| 24 | 35 | 1.12 | 0.93 | 1.02 | Diploid |
| 25 | 36 | 1.13 | 0.94 | 1.02 | Diploid |
| 26 | 37 | 1.12 | 0.93 | 1.02 | Diploid |
| 27 | 39 | 1.10 | 0.91 | 1.00 | Diploid |
| 28 | 43 | 1.13 | 0.94 | 1.02 | Diploid |
| 29 | 44 | 1.05 | 0.88 | 0.96 | Diploid |
| 30 | 48 | 1.13 | 0.94 | 1.03 | Diploid |
| 31 | 49 | 1.11 | 0.92 | 1.01 | Diploid |
| 32 | 53 | 0.96 | 0.80 | 0.87 | Diploid |
| 33 | 58 | 1.07 | 0.89 | 0.97 | Diploid |
| 34 | 66 | 1.05 | 0.87 | 0.95 | Diploid |
| 35 | 67 | 1.06 | 0.88 | 0.96 | Diploid |
| 36 | 68 | 1.05 | 0.87 | 0.95 | Diploid |
| 37 | 1 | 2.34 | 2.01 | 2.16 | Tetraploid |
| 38 | 4 | 2.28 | 1.96 | 2.11 | Tetraploid |
| 39 | 8 | 2.28 | 1.96 | 2.11 | Tetraploid |
| 40 | 17 | 2.26 | 1.94 | 2.09 | Tetraploid |
| 41 | 20 | 2.30 | 1.99 | 2.13 | Tetraploid |
| 42 | 21 | 2.26 | 1.95 | 2.09 | Tetraploid |
| 43 | 28 | 2.36 | 2.03 | 2.18 | Tetraploid |
| 44 | 29 | 2.28 | 1.96 | 2.11 | Tetraploid |
| 45 | 40 | 2.12 | 1.76 | 1.92 | Tetraploid |
| 46 | 50 | 2.15 | 1.78 | 1.95 | Tetraploid |
| 47 | 54 | 2.00 | 1.67 | 1.82 | Tetraploid |
| 48 | 55 | 1.95 | 1.62 | 1.77 | Tetraploid |
| 49 | 56 | 1.94 | 1.62 | 1.76 | Tetraploid |
| 50 | 60 | 2.33 | 1.94 | 2.12 | Tetraploid |
| 51 | 65 | 2.18 | 1.81 | 1.98 | Tetraploid |
| 52 | 69 | 1.97 | 1.64 | 1.79 | Tetraploid |
| 53 | 57 | 4.97 | 4.13 | 4.51 | Octoploid |
| 54 | 61 | 2.85 | 2.37 | 2.59 | Hexaploid |
| 55 | 62 | 2.56 | 2.13 | 2.32 | Hexaploid |
| No. | Plant ID | Relative ploidy  to PL9-4X | Relative ploidy  to WK21-4X | Relative ploidy  (Average) | Estimated ploidy |
| 56 | 100 | 0.25 | 0.27 | 0.26 | Haploid |
| 57 | 125 | 0.23 | 0.25 | 0.24 | Haploid |
| 58 | 126 | 0.24 | 0.27 | 0.26 | Haploid |
| 59 | 149 | 0.25 | 0.28 | 0.26 | Haploid |
| 60 | 150 | 0.25 | 0.27 | 0.26 | Haploid |
| 61 | 81 | 0.49 | 0.55 | 0.52 | Diploid |
| 62 | 84 | 0.44 | 0.50 | 0.47 | Diploid |
| 63 | 86 | 0.47 | 0.53 | 0.50 | Diploid |
| 64 | 94 | 0.45 | 0.50 | 0.48 | Diploid |
| 65 | 104 | 0.40 | 0.44 | 0.42 | Diploid |
| 66 | 105 | 0.44 | 0.49 | 0.46 | Diploid |
| 67 | 111 | 0.49 | 0.54 | 0.51 | Diploid |
| 68 | 114 | 0.49 | 0.53 | 0.51 | Diploid |
| 69 | 115 | 0.49 | 0.54 | 0.52 | Diploid |
| 70 | 117 | 0.48 | 0.53 | 0.50 | Diploid |
| 71 | 118 | 0.45 | 0.48 | 0.47 | Diploid |
| 72 | 119 | 0.38 | 0.42 | 0.40 | Diploid |
| 73 | 121 | 0.47 | 0.52 | 0.49 | Diploid |
| 74 | 122 | 0.47 | 0.52 | 0.50 | Diploid |
| 75 | 123 | 0.47 | 0.52 | 0.49 | Diploid |
| 76 | 124 | 0.49 | 0.52 | 0.50 | Diploid |
| 77 | 128 | 0.49 | 0.55 | 0.52 | Diploid |
| 78 | 129 | 0.48 | 0.53 | 0.50 | Diploid |
| 79 | 130 | 0.47 | 0.53 | 0.50 | Diploid |
| 80 | 131 | 0.42 | 0.47 | 0.45 | Diploid |
| 81 | 132 | 0.44 | 0.48 | 0.46 | Diploid |
| 82 | 133 | 0.45 | 0.48 | 0.46 | Diploid |
| 83 | 139 | 0.47 | 0.52 | 0.49 | Diploid |
| 84 | 141 | 0.50 | 0.53 | 0.52 | Diploid |
| 85 | 146 | 0.47 | 0.50 | 0.49 | Diploid |
| 86 | 147 | 0.50 | 0.53 | 0.52 | Diploid |
| 87 | 151 | 0.47 | 0.52 | 0.49 | Diploid |
| 88 | 152 | 0.49 | 0.55 | 0.52 | Diploid |
| 89 | 153 | 0.47 | 0.53 | 0.50 | Diploid |
| 90 | 155 | 0.48 | 0.54 | 0.51 | Diploid |
| 91 | 158 | 0.47 | 0.50 | 0.48 | Diploid |
| 92 | 160 | 0.44 | 0.49 | 0.47 | Diploid |
| 93 | 175 | 0.45 | 0.50 | 0.48 | Diploid |
| 94 | 180 | 0.50 | 0.55 | 0.52 | Diploid |
| 95 | 181 | 0.44 | 0.47 | 0.46 | Diploid |
| 96 | 185 | 0.46 | 0.52 | 0.49 | Diploid |
| 97 | 188 | 0.49 | 0.54 | 0.51 | Diploid |
| 98 | 201 | 0.40 | 0.44 | 0.42 | Diploid |
| 99 | 195 | 0.99 | 1.11 | 1.05 | Tetraploid |
| 100 | 112 | 0.75 | 0.84 | 0.79 | Triploid |
| 101 | 116 | 0.68 | 0.75 | 0.72 | Triploid |
| 102 | 197 | 0.73 | 0.81 | 0.77 | Triploid |

| Table S4. DNA markers used for genotyping of the AC_1_ and DH_1_ plants. | | | | |
| --- | --- | --- | --- | --- |
| Primer | Chr | Location (Mb, IRGSP1.0 | Forward sequence | Reverce sequence |
| UH1-d2 | 1 | 17.1 | AGGTTTAGCGTCAGTACTTGGG | ACACGGTTGAATGATAGACACT |
| UH2-u4 | 2 | 13.5 | AGTTAAAAGTTAACTCGCCGACG | CAGTAAGCATCCTACGGCTCC |
| UH3-d1 | 3 | 19.9 | TGCTGTGAGAATATGTCCACCA | AGCAAGGGAAGCTATTTACGGA |
| UH4-d1 | 4 | 9.3 | TGTATGGTATTCACCCCCGG | TGGAGCTCTGAGAAATACGAATCC |
| c5-1.25 | 5 | 1.3 | CATGATATCAGTAGGATCACACTTTGT | AAAAATTTACGGCGTAAAAATGATGT |
| UH5-u1 | 5 | 12.2 | GACAGACTAGCGAACTTTGGT | ACCTGCTGTCTCGATTACATCC |
| c5-24.9 | 5 | 24.9 | GGCACTCGCACTTGTTCAAC | ACCTCTCGATACCCATGGTACT |
| UH6-u2 | 6 | 14.9 | ACCAGTTTTTCCATCTCCAAACC | CGGCCGGCTAGAAACAAGAT |
| UH7-u2 | 7 | 11.9 | TCACCGCGGAGAATCAACAA | AGCCATACCAGCTGATGTCG |
| c7-10.2 | 7 | 10.2 | TGAACACTTCCCACAGCTCA | AGGCTGGAGCAGTTTCTCAC |
| UH8-d1 | 8 | 13.7 | TGATCATCACAAGTGTCTTTTCGT | TCCTCATCGGTCAAAGTGCT |
| c8-1.7 | 8 | 1.7 | TACTCCCTCCCCGTCCAAAA | TGAGCATCTTCAGCATCTAATGGA |
| c8-26.4 | 8 | 26.4 | TGCACATACTAGTGATGTAAGCT | CACATCATCACGAGTGGGACA |
| UH9-d1 | 9 | 2.9 | GTGACAGCCAAGTCCGTCAT | GGTCAAGAAGGTGCAGGTGA |
| c9-19.9 | 9 | 19.9 | TGGCTGCTCTTGCACTTATCA | CCCTTTCCAATGGAAGAGAAGGA |
| UH10-d3 | 10 | 8.5 | ACCGAGCACTTATCTACTTGCA | GGGGATGATTTCACCCGTAG |
| c10-4.9 | 10 | 4.9 | AGTACGTGCGCTAGTTTCTGA | TGGATGAGTGATGCATGTATACA |
| c10-12.8 | 10 | 12.9 | TCCCGTGTTATGTCAAGTACAGG | AGCTCATCATCACTGGCCAA |
| c10-22.2 | 10 | 22.2 | GCAGCAACTGATCGTGCAAA | TGGCTAATCCCTCCACAAGG |
| UH11-d1 | 11 | 12.2 | GCTGAAAGTGAAATGTATTGCACC | ACTAGAACGATACTATAACCCGGT |
| c11-5.0 | 11 | 5.0 | TCAACAACTGCAAATTACTGGCA | TGACAAGAACCCTGACAACCA |
| c11-25.0 | 11 | 25.0 | GTCTGTGTGGATGGGCTGAT | TCTGACAACAGCAAACTCGC |
| UH12-d1 | 12 | 12.1 | TGATAGAGCCATGGTGACCG | TGTTGCAAATATTTTGAAGCGATAAG |
| c12-2.0 | 12 | 2.0 | AGAAGAATGCCGAGGAAAACA | CCTGGAGTGCAGTACGATCA |
| c12-25.1 | 12 | 25.1 | CCCATCGGAGTCTTATCAAGGT | AAGGACGTGCAAGCTCGAAG |
| c1-7.38 | 1 | 7.38 | CCATCACCGGGCAGACTCT | GGTGCATATTTCCTCTGATTTTTGC |
| c1-7.42 | 1 | 7.42 | TAGTCCATCAAAGGCTCGCC | GGCATACACCCAGGTATTGT |
| c12-11.91 | 12 | 11.91 | TGAGGCAAGGGCGAGGAG | TCTCAGCATTTTTCATGTGATCGA |

| Table S5 (1/2). Genotyping result of the DH candidates derived from anther culture of the AC_1_ plants. | | | | | | | | | | | | | |
| --- | --- | --- | --- | --- | --- | --- | --- | --- | --- | --- | --- | --- | --- |
| Plant material | Generation | DNA marker | | | | | | | | | | | |
|  |  | UH1-d2 | UH2-u4 | UH3-d1 | UH4-d1 | UH5-u1 | UH6-u2 | UH7-u2 | UH8-d1 | UH9-d1 | UH10-d3 | UH11-d1 | UH12-d1 |
| PL9 | Parent | *S* | *S* | *S* | *S* | *S* | *S* | *S* | *S* | *S* | *S* | *S* | *S* |
| WK21 | Parent | *G* | *G* | *G* | *G* | *G* | *G* | *G* | *G* | *G* | *G* | *G* | *G* |
| WK21-2X/PL9-2X F_1_ | F_1_ | *H* | *H* | *H* | *H* | *H* | *H* | *H* | *H* | *H* | *H* | *H* | *H* |
| 12 | DH  candidate | *S* | *S* | *S* | *S* | *S* | *S* | *S* | *S* | *G* | *S* | *G* | *S* |
| 66 |  | *S* | *G* | *G* | *S* | *S* | *S* | *G* | *G* | *S* | *G* | *G* | *G* |
| 81 |  | *S* | *S* | *G* | *S* | *S* | *S* | *S* | *S* | *G* | *S* | *G* | *S* |
| 84 |  | *S* | *S* | *G* | *G* | *S* | *S* | *G* | *S* | *S* | *G* | *G* | *S* |
| 86 |  | *S* | *G* | *G* | *S* | *S* | *S* | *S* | *S* | *S* | *S* | *G* | *G* |
| 94 |  | *S* | *S* | *G* | *S* | *S* | *S* | *S* | *S* | *S* | *S* | *S* | *S* |
| 98 |  | *S* | *-* | *G* | *S* | *S* | *S* | *S* | *S* | *G* | *G* | *S* | *S* |
| 104 |  | *S* | *G* | *G* | *S* | *S* | *S* | *S* | *S* | *G* | *G* | *S* | *S* |
| 105 |  | *S* | *G* | *G* | *S* | *S* | *S* | *S* | *S* | *G* | *G* | *S* | *S* |
| 111 |  | *S* | *S* | *S* | *G* | *S* | *G* | *S* | *S* | *G* | *S* | *S* | *S* |
| 114 |  | *S* | *G* | *S* | *S* | *G* | *G* | *G* | *G* | *G* | *G* | *G* | *S* |
| 115 |  | *S* | *S* | *S* | *S* | *G* | *G* | *G* | *S* | *S* | *G* | *S* | *S* |
| 117 |  | *S* | *S* | *G* | *S* | *S* | *S* | *G* | *S* | *S* | *S* | *-* | *S* |
| 118 |  | *S* | *S* | *G* | *S* | *S* | *S* | *G* | *S* | *S* | *S* | *G* | *S* |
| 119 |  | *S* | *G* | *G* | *S* | *S* | *S* | *G* | *S* | *S* | *G* | *G* | *S* |
| 121 |  | *S* | *S* | *G* | *S* | *S* | *S* | *S* | *S* | *S* | *G* | *S* | *G* |
| 122 |  | *S* | *S* | *G* | *S* | *S* | *S* | *G* | *G* | *G* | *G* | *G* | *S* |
| 123 |  | *S* | *S* | *G* | *S* | *S* | *S* | *G* | *G* | *G* | *G* | *G* | *S* |
| 124 |  | *S* | *S* | *G* | *S* | *S* | *S* | *G* | *G* | *G* | *G* | *G* | *S* |
| 128 |  | *S* | *S* | *G* | *S* | *S* | *S* | *G* | *S* | *S* | *G* | *G* | *S* |
| 129 |  | *S* | *S* | *G* | *S* | *S* | *S* | *G* | *S* | *S* | *G* | *G* | *S* |
| 130 |  | *S* | *G* | *G* | *S* | *S* | *S* | *S* | *S* | *G* | *S* | *G* | *S* |
| 131 |  | *S* | *S* | *G* | *S* | *S* | *S* | *G* | *G* | *G* | *G* | *G* | *S* |
| 132 |  | *S* | *S* | *G* | *S* | *S* | *S* | *G* | *S* | *G* | *G* | *S* | *G* |
| 133 |  | *S* | *S* | *G* | *S* | *S* | *S* | *G* | *S* | *G* | *-* | *S* | *S* |
| 139 |  | *S* | *-* | *G* | *S* | *S* | *S* | *G* | *G* | *S* | *-* | *G* | *G* |
| 146 |  | *S* | *G* | *G* | *S* | *S* | *S* | *S* | *G* | *G* | *S* | *G* | *G* |
| 147 |  | *S* | *G* | *G* | *S* | *S* | *S* | *S* | *G* | *G* | *S* | *G* | *G* |
| 155 |  | *S* | *S* | *G* | *S* | *S* | *G* | *S* | *S* | *S* | *G* | *G* | *G* |
| 158 |  | *S* | *G* | *G* | *S* | *S* | *S* | *S* | *S* | *G* | *G* | *G* | *G* |
| 160 |  | *-* | *S* | *G* | *S* | *S* | *S* | *G* | *G* | *G* | *G* | *G* | *S* |
| 175 |  | *S* | *G* | *S* | *S* | *G* | *S* | *S* | *G* | *G* | *G* | *-* | *S* |
| 180 |  | *S* | *-* | *G* | *S* | *S* | *S* | *G* | *S* | *S* | *S* | *-* | *S* |
| 201 |  | *S* | *S* | *G* | *S* | *-* | *S* | *G* | *G* | *S* | *S* | *G* | *G* |
| 141 |  | *H* | *G* | *H* | *H* | *S* | *G* | *G* | *S* | *H* | *H* | *H* | *H* |
| 151 |  | *S* | *S* | *S* | *S* | *S* | *S* | *S* | *S* | *G* | *S* | *G* | *S* |
| 152 |  | *S* | *S* | *S* | *S* | *S* | *S* | *S* | *S* | *G* | *S* | *G* | *S* |
| 153 |  | *S* | *S* | *S* | *S* | *S* | *S* | *S* | *S* | *G* | *S* | *G* | *S* |
| 181 |  | *S* | *S* | *G* | *S* | *H* | *S* | *H* | *S* | *S* | *H* | *G* | *S* |
| 185 |  | *S* | *G* | *H* | *H* | *S* | *G* | *G* | *S* | *H* | *H* | *H* | *H* |
| 188 |  | *S* | *G* | *H* | *H* | *S* | *G* | *G* | *S* | *H* | *H* | *H* | *H* |

| Table S5 (2/2). Genotyping result of the DH candidates derived from anther culture of the AC_1_ plants. | | | | | | | | | | | | | | |
| --- | --- | --- | --- | --- | --- | --- | --- | --- | --- | --- | --- | --- | --- | --- |
| Plant material | Generation | DNA marker | | | | | | | | | | | | Decision |
|  |  | c5-24.9 | c7-10.2 | c8-1.7 | c8-26.4 | c9-19.9 | c10-4.9 | c10-12.8 | c10-22.2 | c11-5.0 | c11-25.0 | c12-2.0 | c12-25.1 |  |
| PL9 | Parent | *S* | *S* | *S* | *S* | *S* | *S* | *S* | *S* | *S* | *S* | *S* | *S* | - |
| WK21 | Parent | *G* | *G* | *G* | *G* | *G* | *G* | *G* | *G* | *G* | *G* | *G* | *G* | - |
| WK21-2X/PL9-2X F_1_ | F_1_ | *H* | *H* | *H* | *H* | *H* | *H* | *H* | *H* | *H* | *H* | *H* | *H* | - |
| 12 | DH  candidate | *G* | *S* | *S* | *S* | *G* | *S* | *S* | *S* | *G* | *S* | *S* | *S* | DH |
| 66 |  | *G* | *G* | *S* | *S* | *S* | *G* | *G* | *S* | *G* | *G* | *S* | *G* | DH |
| 81 |  | *S* | *S* | *G* | *S* | *S* | *S* | *S* | *S* | *S* | *G* | *S* | *S* | DH |
| 84 |  | *S* | *G* | *S* | *S* | *S* | *G* | *S* | *S* | *G* | *G* | *S* | *S* | DH |
| 86 |  | *S* | *S* | *G* | *S* | *G* | *S* | *S* | *G* | *G* | *G* | *S* | *G* | DH |
| 94 |  | *S* | *S* | *S* | *S* | *G* | *S* | *S* | *S* | *G* | *G* | *S* | *S* | DH |
| 98 |  | *S* | *S* | *G* | *S* | *G* | *G* | *G* | *G* | *G* | *G* | *S* | *S* | DH |
| 104 |  | *S* | *S* | *G* | *S* | *G* | *G* | *G* | *G* | *G* | *G* | *S* | *S* | DH |
| 105 |  | *S* | *S* | *G* | *S* | *G* | *G* | *G* | *G* | *G* | *G* | *S* | *S* | DH |
| 111 |  | *S* | *S* | *S* | *S* | *G* | *S* | *S* | *S* | *S* | *S* | *S* | *S* | DH |
| 114 |  | *G* | *-* | *G* | *G* | *G* | *G* | *G* | *G* | *G* | *S* | *S* | *G* | DH |
| 115 |  | *G* | *G* | *S* | *S* | *S* | *G* | *G* | *G* | *S* | *S* | *S* | *G* | DH |
| 117 |  | *S* | *G* | *G* | *S* | *S* | *S* | *S* | *S* | *G* | *G* | *S* | *G* | DH |
| 118 |  | *S* | *G* | *G* | *S* | *S* | *S* | *S* | *S* | *G* | *G* | *S* | *G* | DH |
| 119 |  | *S* | *G* | *S* | *S* | *S* | *G* | *G* | *G* | *G* | *G* | *G* | *S* | DH |
| 121 |  | *S* | *S* | *S* | *S* | *G* | *G* | *S* | *S* | *S* | *S* | *G* | *S* | DH |
| 122 |  | *G* | *G* | *G* | *S* | *G* | *G* | *G* | *G* | *G* | *G* | *S* | *G* | DH |
| 123 |  | *G* | *G* | *G* | *S* | *G* | *G* | *G* | *G* | *G* | *G* | *S* | *G* | DH |
| 124 |  | *G* | *G* | *G* | *S* | *G* | *G* | *G* | *G* | *G* | *G* | *S* | *G* | DH |
| 128 |  | *S* | *G* | *S* | *S* | *S* | *G* | *G* | *G* | *G* | *S* | *S* | *S* | DH |
| 129 |  | *S* | *G* | *S* | *S* | *S* | *G* | *G* | *G* | *G* | *S* | *S* | *S* | DH |
| 130 |  | *S* | *S* | *G* | *S* | *-* | *S* | *S* | *S* | *G* | *S* | *G* | *G* | DH |
| 131 |  | *G* | *G* | *G* | *S* | *G* | *G* | *G* | *G* | *G* | *G* | *S* | *G* | DH |
| 132 |  | *S* | *G* | *S* | *S* | *S* | *G* | *G* | *G* | *S* | *S* | *G* | *S* | DH |
| 133 |  | *S* | *G* | *S* | *S* | *S* | *G* | *S* | *S* | *S* | *G* | *G* | *G* | DH |
| 139 |  | *S* | *G* | *G* | *S* | *S* | *S* | *G* | *S* | *G* | *G* | *G* | *S* | DH |
| 146 |  | *G* | *S* | *S* | *S* | *S* | *S* | *S* | *G* | *G* | *G* | *G* | *G* | DH |
| 147 |  | *G* | *S* | *S* | *S* | *S* | *S* | *S* | *G* | *G* | *G* | *G* | *G* | DH |
| 155 |  | *G* | *S* | *G* | *G* | *S* | *G* | *S* | *S* | *G* | *G* | *G* | *S* | DH |
| 158 |  | *S* | *S* | *S* | *S* | *G* | *G* | *G* | *G* | *G* | *G* | *G* | *G* | DH |
| 160 |  | *G* | *G* | *G* | *S* | *G* | *G* | *G* | *G* | *G* | *G* | *S* | *G* | DH |
| 175 |  | *G* | *S* | *G* | *G* | *G* | *G* | *G* | *G* | *G* | *G* | *S* | *G* | DH |
| 180 |  | *S* | *G* | *G* | *S* | *S* | *S* | *S* | *S* | *G* | *G* | *S* | *G* | DH |
| 201 |  | *G* | *G* | *S* | *S* | *S* | *S* | *S* | *S* | *G* | *G* | *S* | *G* | DH |
| 141 |  | *H* | *G* | *S* | *S* | *H* | *G* | *H* | *H* | *G* | *H* | *H* | *H* | - |
| 151 |  | *H* | *S* | *H* | *S* | *G* | *S* | *S* | *S* | *H* | *H* | *S* | *S* | - |
| 152 |  | *H* | *S* | *H* | *S* | *G* | *S* | *S* | *S* | *H* | *H* | *S* | *S* | - |
| 153 |  | *H* | *S* | *H* | *S* | *G* | *S* | *S* | *S* | *H* | *H* | *S* | *S* | - |
| 181 |  | *S* | *G* | *G* | *S* | *S* | *S* | *H* | *S* | *G* | *G* | *S* | *G* | - |
| 185 |  | *H* | *G* | *S* | *S* | *H* | *G* | *H* | *H* | *G* | *H* | *H* | *H* | - |
| 188 |  | *H* | *G* | *S* | *S* | *H* | *G* | *H* | *H* | *G* | *H* | *H* | *H* | - |

| Table S6. Preferentially transmitting alleles in each HS locus. | | |
| --- | --- | --- |
| HS locus | Chr. | Preferentially transmitting allele |
| *S1* | 6 | *glaberrima* |
| *S2* | 4 | *sativa* |
| *S19* | 3 | *glaberrima* |
| *S20* | 7 | *glaberrima* |
| *S21* | 7 | *glaberrima* |
| *S29(t)* | 2 | *sativa* |
| *S58* | 1 | *sativa* |

Table S7. Kruskal-Wallis test among DH2 lines.

| CL | PL | PN | CW | PW | HI | TS | TGW | DTH |
| --- | --- | --- | --- | --- | --- | --- | --- | --- |
| 0.002^*^ | 0.005^*^ | 0.160 | 0.059 | 0.004^*^ | 0.002^*^ | 0.001^*^ | 0.005^*^ | 0.001^*^ |
| p<0.05 | | | | | | | | |

Table S8. Pollen and seed fertility of PL9-2X and WK21-2X, and DH materials in the DH_2_ population.

| Plant material | PF | SR |
| --- | --- | --- |
| PL9-2X | 96.9±1.0 | 88.4±4.4 |
| WK21-2X | 91.4±1.7 | 90.6±4.5 |
| DH#81 | 92.8±1.8 | 70.7±6.2 |
| DH#84 | 77.3±0.5 | 55.3±5.2 |
| DH#94 | 83.3±9.4 | 76.6±4.6 |
| DH#98 | 54.8±4.0 | 11.6±2.8 |
| DH#115 | 88.4±5.1 | 89.8±4.1 |
| DH#124 | 74.7±11.4 | 55.9±6.9 |
| DH#129 | 81.5±10.0 | 62.3±10.3 |
| DH#130 | 83.7±7.5 | 85.6±2.2 |
| DH#133 | 72.6±7.8 | 83.8±3.7 |
| DH#201 | 77.2±11.6 | 32.7±3.1 |
